# Inhibition of NETosis via PAD4 alleviated inflammation in giant cell myocarditis

**DOI:** 10.1016/j.isci.2023.107162

**Published:** 2023-06-16

**Authors:** Zhan Hu, Xiumeng Hua, Xiuxue Mo, Yuan Chang, Xiao Chen, Zhenyu Xu, Mengtao Tao, Gang Hu, Jiangping Song

**Affiliations:** 1 Beijing Key Laboratory of Preclinical Research and Evaluation for Cardiovascular Implant Materials, Animal Experimental Centre, Fuwai Hospital, National Centre for Cardiovascular Disease, Chinese Academy of Medical Sciences and Peking Union Medical College, Beijing 100037, China; 2 Department of Cardiovascular Surgery, Fuwai Hospital, National Center for Cardiovascular Diseases, Chinese Academy of Medical Sciences and Peking Union Medical College, Beijing, People’s Republic of China; 3 State Key Laboratory of Cardiovascular Disease, Fuwai Hospital, National Center for Cardiovascular Diseases, Chinese Academy of Medical Sciences and Peking Union Medical College, Beijing 100037, China; 4 The Cardiomyopathy Research Group at Fuwai Hospital, Tianjin 300071, China; 5 School of Statistics and Data Science, LPMC and KLMDASR, Nankai University, Tianjin 300071, China; 6 Department of Pathology Center, State Key Laboratory of Cardiovascular Disease, Fuwai Hospital, National Center for Cardiovascular Diseases, Chinese Academy of Medical Sciences and Peking Union Medical College, Beijing 100037, China

**Keywords:** Cardiovascular medicine, Immunology, Transcriptomics

## Abstract

Giant cell myocarditis (GCM) is a rare, usually rapidly progressive, and potentially fatal disease. Detailed inflammatory responses remain unknown, in particular the formation of multinucleate giant cells. We performed single-cell RNA sequencing analysis on 15,714 *Cd45*^+^ cells extracted from the hearts of GCM rats and normal rats. NETosis has been found to contribute to the GCM process. An inhibitor of NETosis, GSK484, alleviated GCM inflammation *in vivo*. *MPO* (a marker of neutrophils) and *H3cit* (a marker of NETosis) were expressed at higher levels in patients with GCM than in patients with DCM and healthy controls. Imaging mass cytometry analysis revealed that immune cell types within multinucleate giant cells included CD4^+^ T cells, CD8^+^ T cells, neutrophils, and macrophages but not B cells. We elucidated the role of NETosis in GCM pathogenesis, which may serve as a potential therapeutic target in the clinic.

## Highlights

• Single-cell RNA profiles of cardiac immune cells from GCM were first generated
• Neutrophils were reported to participate in the pathogenesis of GCM
• NETosis could be a target, inhibited by GSK484, to alleviate inflammation in GCM
• The expression of *MPO* and *H3cit* was upregulated in the hearts of patients with GCM

## Graphical abstract

**Figure:**
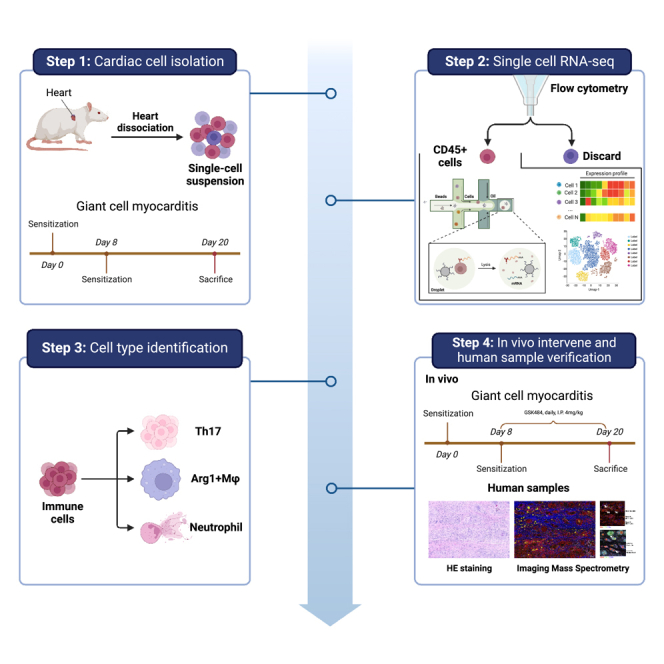

## Introduction

Giant cell myocarditis (GCM) is a rare and highly fatal disease; its typical pathological characteristics include infiltration of inflammatory cells, myocardial necrosis, and multinucleate giant cell formation.^1^ In 1905, Saltykow described and named the first case of GCM, and approximately 100 GCM cases were reported worldwide in the next 100 years.^2^^,^^3^ The development of GCM is rapid, with approximately 2–3 weeks passing from symptom onset to hospitalization, and the major clinical presentations are acute congestive heart failure and ventricular arrhythmia.^4^ Clinically, almost 20% of patients with GCM also present with autoimmune diseases such as inflammatory bowel disease, thyroiditis, celiac disease, and rheumatoid arthritis, and there have been a few patients with thymoma or lymphoma.^5^^,^^6^^,^^7^ One typical characteristic of patients with GCM is multinucleate giant cell formation, which is the basis of GCM diagnosis.^8^ The prognosis of GCM is extremely poor before the usage of immunosuppression, as almost all patients need heart transplantations.^9^ Early treatment with cyclosporin-based immunosuppression, improved the 1-year survival rate of giant cell myocarditis to 90%.^10^ Moreover, the recurrence of GCM after heart transplantation is high; long-term immunosuppressant therapy can reduce the risk of recurrence.^4^^,^^11^

The pathogenesis of GCM is still unclear, but it is considered an autoimmune disease. Animal models indicate that GCM can be induced by autoimmunity, and inflammation cell analysis showed that the major inflammatory cells included macrophages and *Cd4*^+^ T cells.^12^^,^^13^ Lassner D et al. examined gene expression profiles in endomyocardial biopsy samples from 10 patients with histopathologically proven idiopathic giant cell myocarditis, 10 with cardiac sarcoidosis (CS), 18 with active myocarditis, and 80 inflammation-free control subjects by quantitative real-time PCR.^14^ Their findings suggested that genes such as CCR5 and CCR6 related to T cells were expressed at higher levels in patients with GCM, which supports the opinion that patients with GCM suffer from massive T cell infiltration.^14^ The detailed cardiac immunological environment of GCM is unknown. In addition, infiltration of neutrophils in GCM has rarely been reported due to the consensus that macrophages and *Cd4*^+^ T cells are predominant components of infiltrating mononuclear cells in GCM.^8^ Neutrophil extracellular traps (NETs) are widely reported in a large range of inflammatory infectious and noninfectious diseases, and NETs can also activate other immune cells, such as B cells, antigen-presenting cells, and T cells.^15^ The process of NET generation, called NETosis, is a specific type of cell death that differs from necrosis and apoptosis. It was reported that NETosis participated in autoimmunity as an inflammation inducer.^16^ The roles of NETosis in the pathogenesis of GCM are unknown. Currently, a variety of approaches to therapeutically target neutrophils have emerged, including strategies to enhance, inhibit, or restore neutrophil function, with several agents entering clinical trials.^17^

With the advance of single-cell RNA sequencing (scRNA-seq) in cardiovascular diseases such as heart failure,^18^ myocarditis,^19^ and myocardial infarction,^20^ it is possible to investigate the cardiac immune cells of GCM. Investigation of cardiac immune cells in GCM can provide insight into pathogenesis and can help develop new immunotherapy strategies for GCM.

Here, we used a GCM animal model coupled with scRNA-seq to investigate the transcriptional profile of immune cells that mediate the inflammatory response in rat hearts. In this study, we first investigated infiltrating immune cells in GCM at the single-cell level and focused on the role of NETs in GCM. In addition, we applied imaging mass cytometry (IMC) to reveal the formation of multinucleate giant cells that were composed of macrophages and neutrophils. We also found that NETs appeared in clinical samples from patients with GCM. To reveal the roles of NETs, an inhibitor (GSK484) of PAD4, an enzyme required for NET formation,^21^^,^^22^ was applied to treat the GCM model *in vivo*. The inhibition of NET formation alleviated the infiltration of immune cells in GCM. Moreover, NETs can recruit other immune cells, especially macrophages and T cells, via *Ccdc25*. Thus, NETosis may represent a novel therapeutic target for the treatment of GCM.

## Results

### scRNA-seq analysis of the GCM model

The rat GCM model was established according to a previous report,^23^ and the clinicopathological phenotype was evaluated on day 21. The left ventricle end-diastolic dimension (LVEDD) was larger while the left ventricular ejection fraction (LVEF) was lower in the GCM group (LVEDD: 5.08 ± 0.16 vs. 5.82 ± 0.15, p = 0.008; LVEF: 86.70 ± 2.95 vs. 77.60 ± 2.32, p = 0.035; Supplementary material online, Figures S1A–S1D). The classic histopathologic features can be seen in GCM groups, including the presence of multinucleated giant cells, a lymphocytic inflammatory infiltrate, and myocyte necrosis (Supplementary material online, Figure S1E).^24^ Following the histological examination, we pooled 5 hearts from each group to isolate single cells. The chosen GCM rat hearts shared similar immune states.

**Figure 1:**
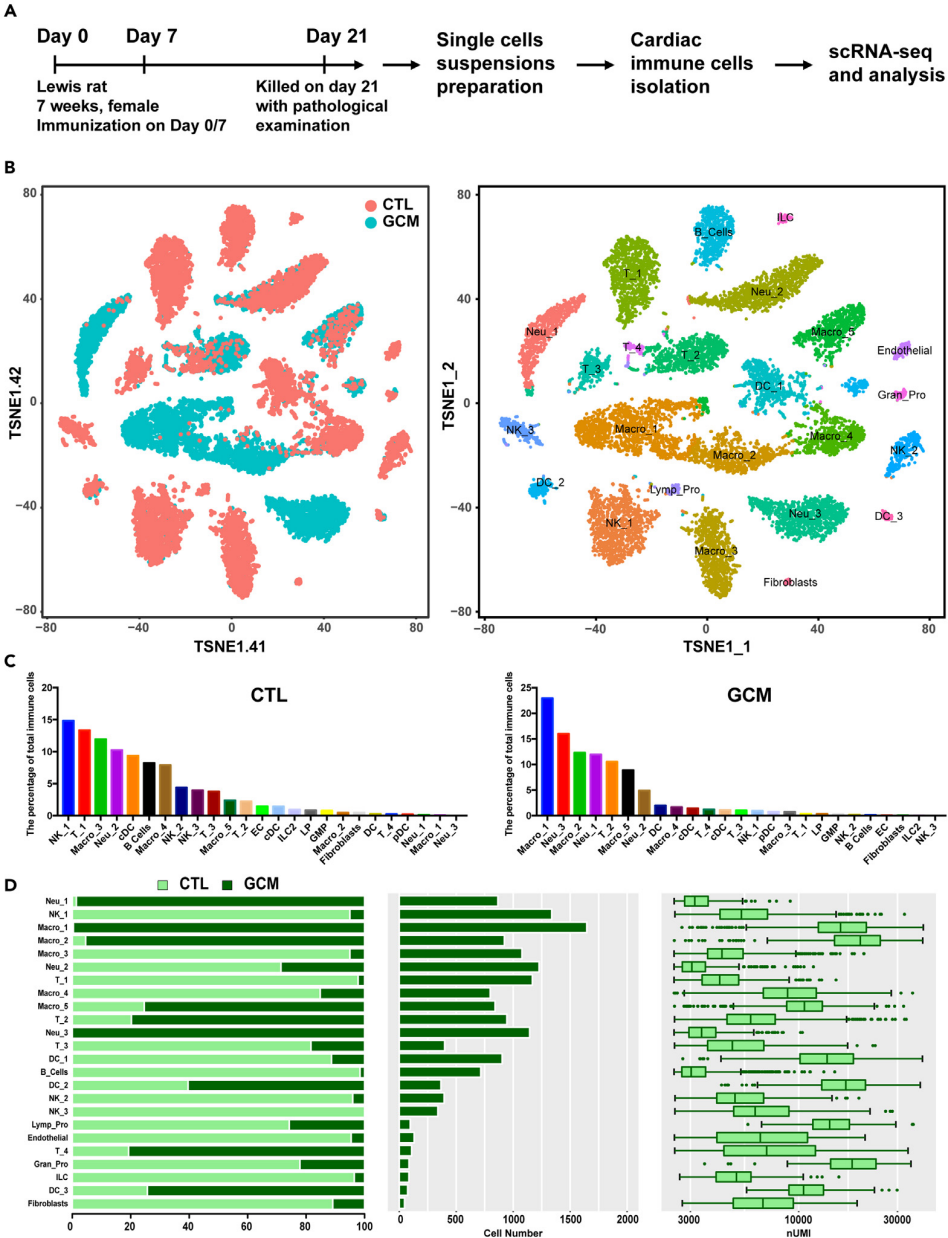
Overview of the 15,714 single cells isolated from the GCM (A) The flowchart of our study includes the study time points, FACS strategy, and scRNA-seq platform. (B) Profiles of the tSNE plots of the 15,714 immune cells, with each cell color-coded (from left to right) for its sample phase of origin and cell cluster. (C) The percentage of each cluster in the control and GCM. D. For each of the 24 cell clusters (from left to right), the fraction of cells originating from controls and GCMs, the number of cells, and boxplots of the number of transcripts are shown to provide an overview of all the immune cells. FACS: flow cytometry and fluorescence-activated cell sorting; GCM: giant cell myocarditis. See also Figures S1–S4 and Tables S1 andS2.

The harvested cardiac *Cd45*^+^ cells from GCM and normal controls were sequenced on a 10x Genomics platform (Figure 1A; Supplementary material online, Figure S2). A total of 15,714 cells were included in the subsequent analysis after filtering was performed (STAR Methods; Supplementary material online, Figure S3 and Table S1). We identified macrophages (5 cell clusters), neutrophils (3 cell clusters), T cells (4 cell clusters), natural killer cells (NK, 3 cell clusters), dendritic cells (DCs, 4 cell clusters), B cells (1 cell cluster), endothelial cells (1 cell cluster), lymphoid progenitors (1 cell cluster), granulocyte-macrophage progenitors (1 cell cluster), group 2 innate lymphoid cells (1 cell cluster), and fibroblasts (FB, 1 cell cluster) (Figure 1B; Supplementary material online, Table S2 and Figure S4). The immunological constitution was completely different between the two groups. The top 5 immune cell clusters were NK cluster 1, T cell cluster 1, macrophage cluster 3, neutrophils cluster 2, and cDCs in the control group, while the top 5 immune cell clusters were macrophage cluster 1, neutrophil cluster 3, macrophage cluster 2, neutrophil cluster 1, and T cell cluster 2 (Figure 1C). Each cell cluster was derived from different phases and had different cell numbers and transcriptional activities, as determined by unique molecular identifiers (Figure 1D; Supplementary material online, Table S2). Some immune cell clusters were derived from different groups, indicating that these cell clusters may contribute to certain pathophysiological processes.

### Two specific macrophage clusters involved in GCM have roles in phagocytosis

**Figure 2:**
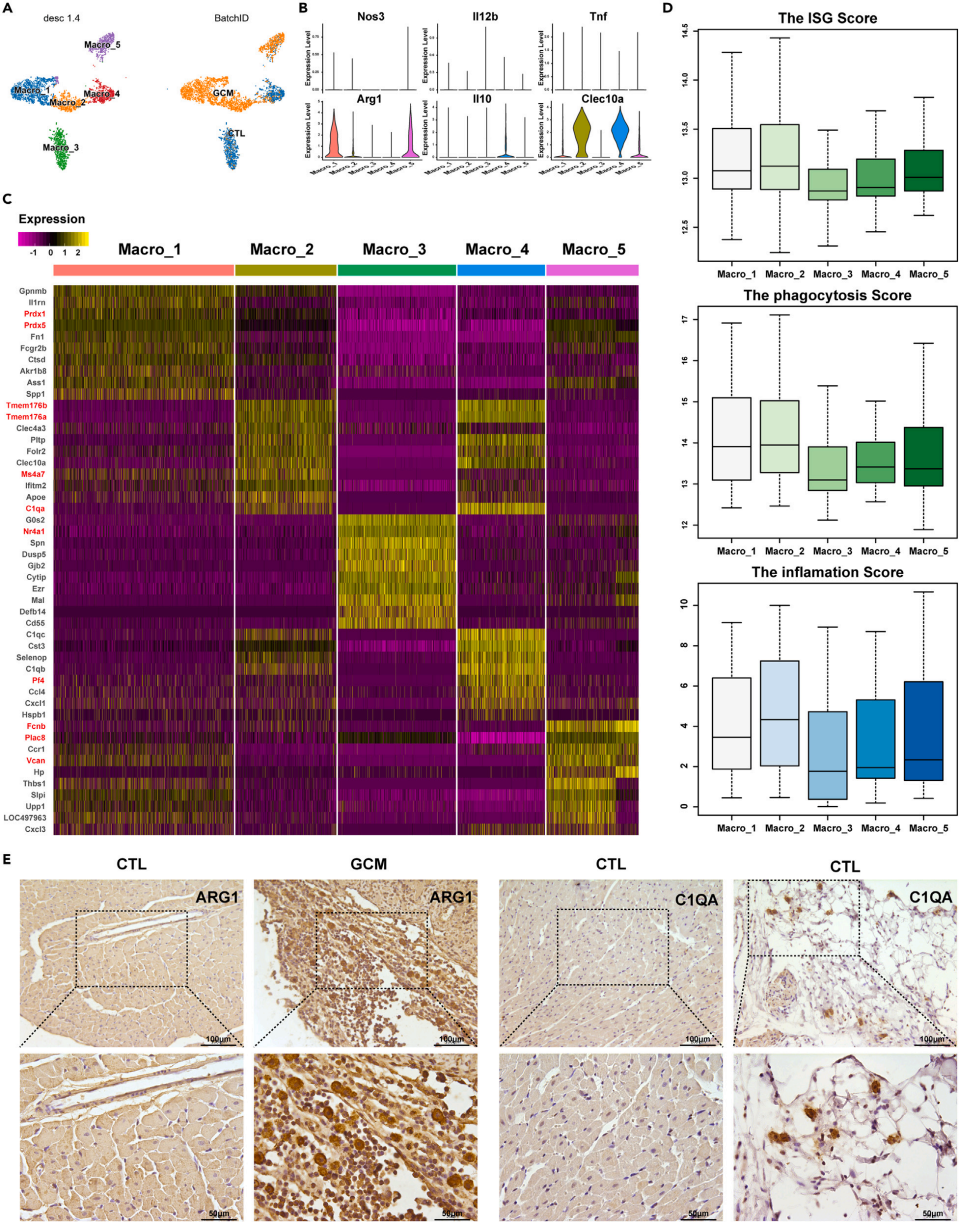
Macrophage cell clusters (A) The tSNE plots of 5,284 macrophages were color-coded according to their associated cluster (left panel) and the sample type of origin (right panel). (B) Violin plots showing the M1/2 marker genes in each macrophage cluster to identify the polarization type. (C) Heatmap of DEGs in each cell cluster. (D) The scores of IFN-γ-stimulated genes, phagocytosis, and inflammation in each cluster. E. The IHC results of ARG1 and C1QA in the control and GCM groups. DEGs: differentially expressed genes. See also Figures S5, S9 and Tables S3, S6.

Macrophages were reported as the major immune cells that make up giant cells.^8^ In this study, 5,284 macrophages were detected as the largest cell population and were clustered into 5 clusters (Figure 2A). According to the macrophage distribution, macrophage clusters 1 and 2 were almost all derived from GCM, indicating that these two cell clusters may be related to GCM (Figure 2A). The macrophage polarization pattern is vital in macrophage biology^25^ (Figure 2B); in this case, classical macrophage polarization was not suitable to study the polarization patterns of macrophages at single-cell resolution.^26^ The gene expression of all 5 clusters was distinct (Figure 2C). *Prdx1* and *Prdx5* were the hallmark genes of macrophage cluster 1 (Figure 2C), and these genes were reported to be associated with autoimmunity.^27^^,^^28^ In addition, macrophage cluster 1 expressed arginine synthesis-associated genes such as *Arg1* and *Ass1.**^29^* Arginine, whose homeostasis is severely disturbed and can be triggered by infiltrating neutrophils as well as bacterial components,^29^ supports continual efferocytosis in macrophages.^30^ Macrophage cluster 2 expressed *Ms4a* family genes such as *Ms4a7*, *Tmem176a*, and *Tmem176b* (Figure 2C). In addition, macrophage cluster 3, the major macrophage cluster under normal conditions, expressed *Nr4a1*, which inhibited the polarization of macrophages toward a proinflammatory M1 phenotype.^31^ Macrophage cluster 4 expressed *Pf4*, a pleiotropic inflammatory chemokine, which limited the activation of resident macrophages.^32^ Macrophage cluster 5 was characterized by the expression of *Fcnb*, *Plac8* (*Onzin*), and *Vcan* (Figure 2C). Macrophage cluster 1/2 had high scores for interferon-stimulated genes, phagocytosis, and inflammation (Figure 2D; Supplementary material online, Table S3). The immunohistochemistry (IHC) results showed that macrophage clusters 1 (ARG1^+^ macrophages) and 2 (C1QA^+^ macrophages) were the hallmark macrophage clusters of GCM. Cardiac macrophages consist of tissue-resident cells and recruited monocyte-derived macrophages from the systemic circulation.^33^ Macrophage cluster 1 was monocyte-derived macrophages, and macrophage cluster 2 was mixed macrophages according to the expression of the marker gene (monocyte-derived macrophages: *Gpnmb**^34^**, Ly6c*; resident macrophages: *Mrc1*, *C1qa*; Supplementary material online, Figure S5).

### Th17 cell activation in the GCM

**Figure 3:**
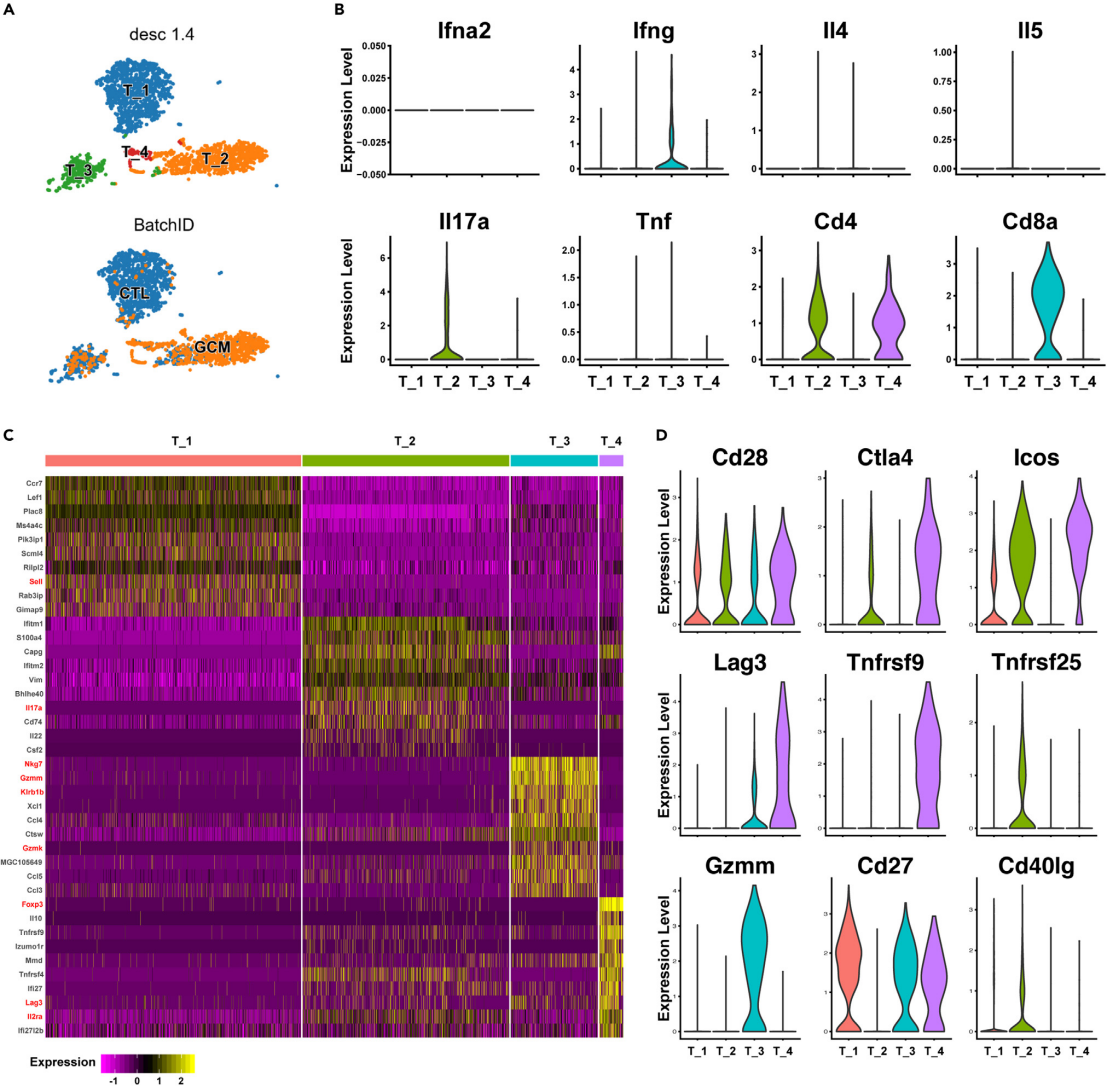
T cell clusters in GCM (A) The tSNE plots of 2,619 T cells are color-coded as described in Figure 2A. (B) Violin plots showing Th1 cytokines, Th2 cytokines, Th17 cytokines, and *Cd4* and *Cd8* genes in T cell clusters. (C) Heatmap of DEGs in each T cell population. (D) Different activation factor expression in the T cell subcluster.

A total of 2,619 T cells were detected and clustered into 4 clusters (Figure 3A). Although GCM has been reported as a T cell-mediated autoimmune disease,^8^ no reports have investigated the details of the infiltration of T lymphocytes. T helper (Th) cells are the major effector T cells,^25^ so we investigated Th-related cytokine expression in T cell clusters (Figure 3B). Th1 cytokines (*Ifna*, *Ifng*) were expressed at low levels in all four T cell clusters, Th2 cytokines (*Il4*, *Il5*) were not detected in T cell clusters, and Th17 cytokines (*Il17*) were highly expressed in T cell cluster 2 (Figure 3B). According to the important surface marker (*Cd4*, *Cd8*), cluster 3 T cells were cytotoxic T lymphocytes (CTLs), while cluster 2 T cells were Th17 cells (Figure 3B). In the GCM, CD4^+^ T cells were the majority of the total T cell population (more than 90%), which may explain why researchers agree that *Cd4*^+^ T cells are the major T cell population in GCM.^35^ In this study, we found that Th17 cells were the major T cell population of GCM (more than 80%), which was the first report to reveal the detailed T cell population of GCM at the single-cell level. There were different gene expression patterns in T cell clusters (Figure 3C). T cell cluster 1 was *Ccr7*^+^*Lef1*^+^*Sell*^+^ T cells, which belonged to central memory T cells.^36^ T cell cluster 2 was characterized by the expression of *Il17a*, *Il22*, and *Cd74*. T cell cluster 3 expressed cytotoxicity factors such as *Nkg7*, *Gzmm*, *Klrb1b*, and *Gzmk*, which suggested that these cluster cells were cytotoxic T cells. T cell cluster 4 was *Cd4*^+^*Il2ra* (*Cd25*) ^+^*Foxp*3^+^ T cells, suggesting regulatory T cells (Treg cells). The mechanism of T cell activation is important in T cell biology,^37^ and we found that different activation factors were detected in different T cell clusters. *Cd27* was detected in T cell cluster 1, CTLs, and Treg cells but not in Th17 cells. *Tnfrsf25* and *Cd40lg* were only expressed in Th17 cells, while *Tnfrsf9* was only expressed in Treg cells. In addition, some activation factors, such as *Ctla4*, *Icos*, and *Lag3*, were expressed at higher levels in Treg cells. For CTLs, *Gzmm* was only expressed in this cluster. Together, under normal conditions, the major T cells were central memory T cells, while Th17 cells were the hallmark T cell population in GCM, which cluster expressed *Cd74* and *Il22*.

### NETosis involved in the pathogenesis of GCM

**Figure 4:**
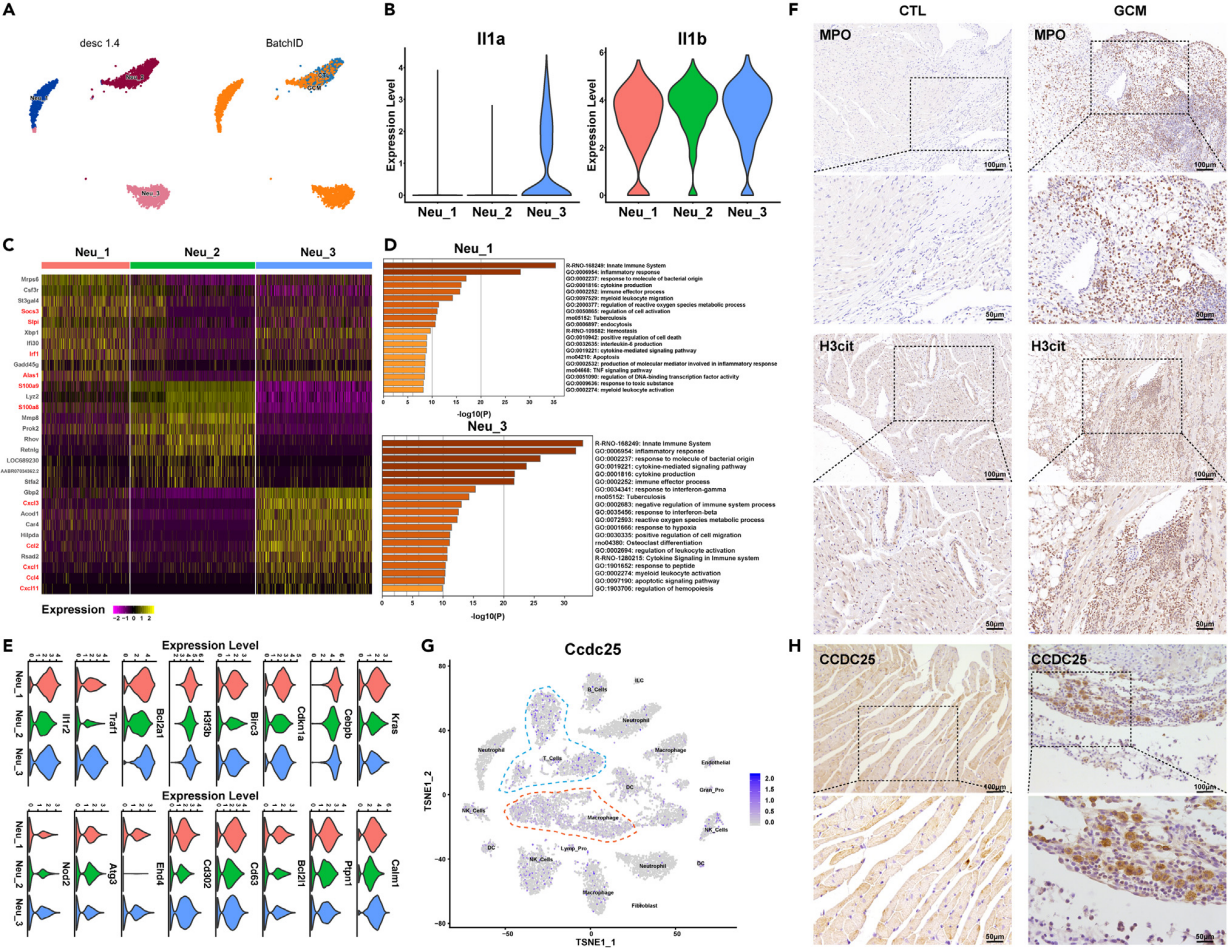
NETosis participates in the pathogenesis of GCM (A) The tSNE plots of 3,237 neutrophils are color-coded as described in Figure 2A. (B) Violin plots showing the expression of *Il-1a* and *Il-1b* in each neutrophil cluster. (C) Heatmap of DEGs in neutrophil clusters. (D) The enrichment pathways of Neu-1 and Neu-3 are hallmarks of GCM. (E) Violin plots of specific marker genes in neutrophil clusters. (F) IHC results of *MPO* and *H3cit* in controls and GCMs. (G) Feature plot of *Ccdc25* in immune cells. (H) IHC results of *CCDC25* in controls and GCMs. See also Figures S6–S7.

The role of neutrophils has always been ignored in GCMs. A total of 3,237 neutrophils were detected and clustered into 3 clusters (Figure 4A). Neutrophils were the second largest cell population of GCM, with 32.86% of the total population from GCM, while neutrophils only accounted for 10.37% of the total population of normal controls (Supplementary material online, Table S2). This result indicated increased neutrophil infiltration into the infiltrates of myocardial rats with GCM. Considering that *Il1* plays a pivotal role in the pathogenesis of myocarditis, we investigated its expression in neutrophils.^38^ As shown in Figure 4B, *Il1β* was expressed in all three clusters, while *Il1α* was only expressed in neutrophil cluster 3. *Il1α* is a potential early trigger of acute inflammation,^39^ so neutrophil cluster 3 may be the trigger of inflammation in GCM. In addition, neutrophil cluster 1 was mostly from GCM, which may be associated with the acute inflammatory response. The three neutrophil clusters shared different gene expression patterns (Figure 4C): neutrophil cluster 1 expressed *Socs3*, *Slpi*, *Irf1*, and *Alas1*; neutrophil cluster 2 expressed *S100a9/8*; and neutrophil cluster 3 expressed *Cxcl3*, *Ccl2*, *Cxc1*, *Ccl4*, and *Cxcl11*. The functions of different neutrophils suggested that NETosis-related pathways were enriched in neutrophil cluster 1, which was the hallmark cell cluster in GCM, such as immune effector process, endocytosis, positive regulation of cell death, and apoptosis (Figures 4A and 4D). Consistent with this finding, the genes related to those pathways were expressed at higher levels in neutrophil cluster 1 (Figure 4E). IHC of *MPO* (a marker of neutrophils) and *H3cit* (a marker of NETosis) showed that NETosis was involved in the GCM process (Figure 4F). Combining the functions of GCM-related neutrophils, including myeloid leukocyte migration and production of molecular mediators involved in the inflammatory response, we speculated that NETosis contributed to the pathogenesis of GCM by recruiting other infiltrating immune cells into the heart, forming multinucleate giant cells. As reported, the transmembrane protein *Ccdc25* is a NET-DNA receptor on cancer cells that senses extracellular DNA and subsequently activates the ILK–β-parvin pathway to enhance cell motility,^40^ so we inferred that NETosis recruited immune cells via *Ccdc25*. To validate this hypothesis, *Ccdc25* was expressed in immune cells in the GCM group, especially macrophage Cluster 1/2 and Th17 cells, and IHC staining was conducted to suggest that the expression of *Ccdc25* was higher in the GCM group (Figure 4H). Taken together, NETosis was involved in the pathogenesis of GCM.

### Intercellular communication network analysis revealed the roles of NETosis in GCM

We performed an intercellular communication network analysis of GCM samples by CellChat.^41^ The biologically significant cell-cell communication network was inferred using CellChat, which assigns each interaction with a probability value and performs a permutation test. From the cell-cell communication network, we found some significant signaling pathways, such as APRIL, BAFF, TWEAK, THY1, THBS, SPP1, SN, IL1, CXCL, and CSF (Supplementary material online, Figure S6).

From the source sets to the target sets (Neu_1, Neu_2, Neu_3, Supplementary material online, Figure S6A), there are significant signaling pathways, such as APRIL, BAFF, TWEAK, THY1, THBS, SPP1, and SN, especially IL-1. Furthermore, IL-1 plays a pivotal role in the pathogenesis of myocarditis.^26^ In addition, the IL1B-IL1R2 pairs were found primarily between macrophage clusters and neutrophil clusters. This suggests that macrophages may induce NETosis, which was consistent with reports that macrophage-derived IL-1β enhances NET formation.^42^^,^^43^

From the source sets (Neu_1, Neu_2, Neu_3) to the target sets (Supplementary material online, Figure S6B), there are significant signaling pathways, such as CXCL and CSF. CSF1-CSF1 receptor (CSF1R) pairs play a pivotal role in the CSF pathway. As reported, M2 polarization is known to be triggered by *CSF1*/*CSF1R* signaling,^44^ supporting that macrophage clusters (Macro_1, Macro_2) could be triggered by infiltrating neutrophils (Neu_3), supporting continual efferocytosis in macrophages. The CXCL signaling pathway indicates that there is communication between neutrophil clusters (Neu_3) and T cells (T_2, T_3, T_4), and CXCR3 is the major receptor in T cells. CXCR3 was reported to guide effector and memory T cell migration to inflammatory lesions and contribute to disease pathogenesis. The expression levels of CXCR3 ligands (*Cxcl9*, *Cxcl10*, and *Cxcl11*) were highest in Neu_3 among neutrophils (Supplementary material online, Figure S7). In addition, these ligands were also expressed in some monocyte-derived macrophage clusters in GCM rats compared to control rats (Supplementary material online, Figures S5 and S7). CXCR3 ligands might attract these T cells, thereby promoting disease.^45^ Together, NETosis may contribute to the pathogenesis of GCM by recruiting other infiltrating immune cells into the heart but with different pairs.

### Inhibition of NETosis in GCM

**Figure 5:**
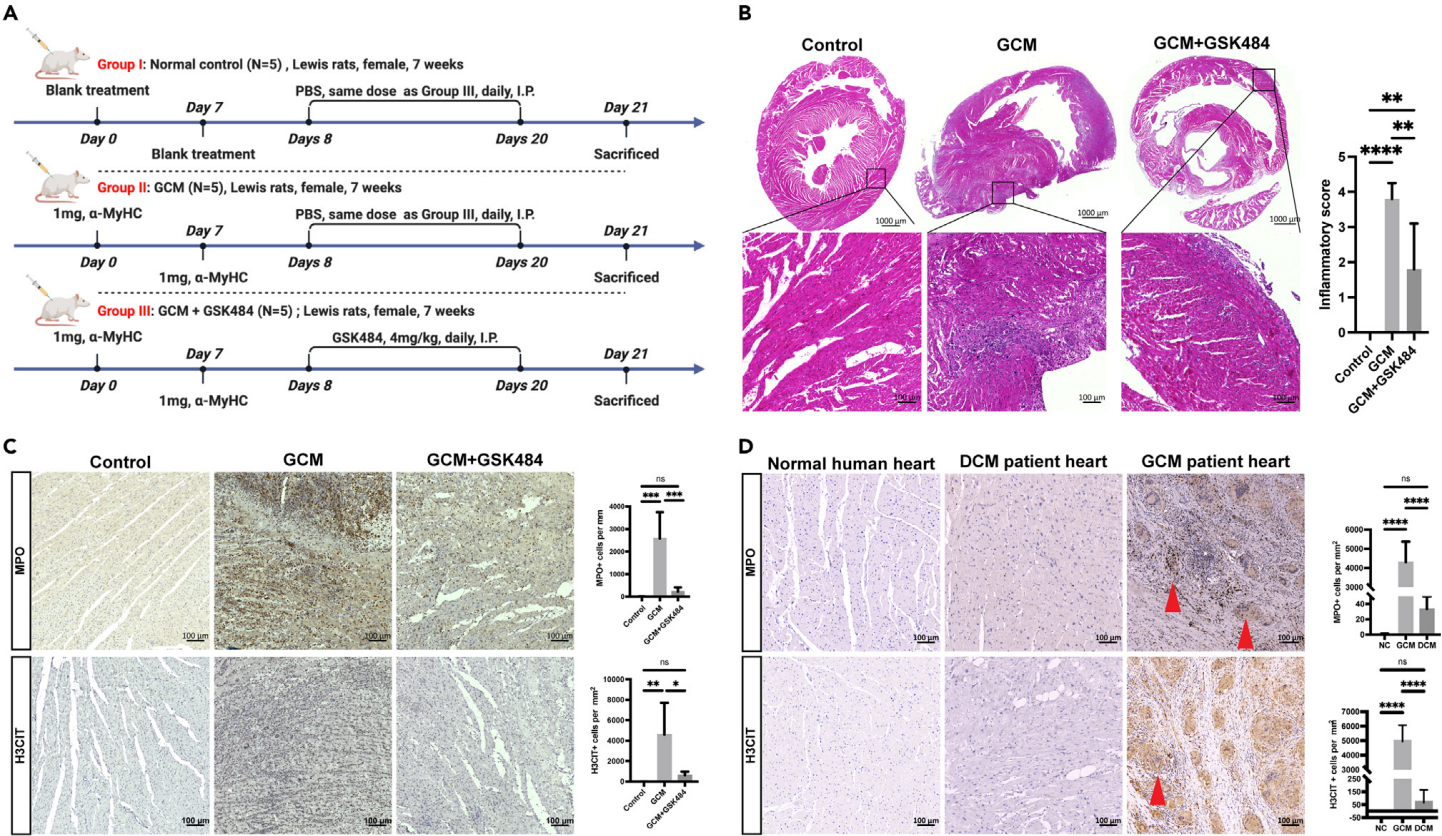
The effect of a NETosis inhibitor on GCM (A) Experimental design of the GSK484 treatment investigation in the GCM model. (B) Representative histological images of the hearts and statistical analysis indicating the summarized inflammatory score of the normal controls, GCM, and GCM + GSK484 samples (n = 5, per group). (C) Representative IHC image and statistical analysis of *MPO* and *H3CIT* of the normal controls, GCM, and GCM + GSK484 samples (n = 5, per group). (D) Representative IHC images and statistical analysis of *MPO* and *H3CIT* in healthy controls, patients with DCM, and patients with GCM. The red arrows indicate multinucleated giant cells. Scale bar: 100 μm n = 5, NS = not significant, ∗p < 0.05; ∗∗p < 0.01; ∗∗∗p < 0.001, ∗∗∗∗p < 0.0001. Data are presented as mean ± SEM. See also Table S4.

As described above, macrophage cluster 1/2 and Th17 cells were the major inflammatory cell clusters of GCM, which expressed *Ccdc25* (Figure 4G), the receptor of NET-DNA. As the roles of neutrophils in the pathogenesis of GCM were reported for the first time, we further investigated the contribution of NETosis to GCM through an inhibitor of PAD4 with a drug, such as GSK484.^22^ Thus, we established another GCM model (n = 10 rats) after two inductions on day 0 and day 7 to investigate the therapeutic effects of GSK484 on GCM (Figure 5A). GSK484 was injected into each GCM rat for 2 weeks (from day 8 to day 20) daily before the rats were sacrificed on day 21. First, the intraperitoneal injection of GSK484 into GCM rats attenuated leukocyte and giant cell accumulation in the hearts and ameliorated inflammation on day 21 (Figure 5B). As described previously, the infiltration of neutrophils in the myocardium of GCM increased significantly, and abundant NETosis was also observed in the myocardium of GCM, with a significant difference on day 21 after two sensitizations according to the IHC staining results (Figure 4, Figure 5). Strikingly, we treated GCM rats with this compound to inhibit PAD4^22^ and observed that NETosis, neutrophil infiltration, and the inflammatory response in the myocardium of GCM were significantly inhibited (Figures 5B and 5C). Taken together, NETosis participates in the pathogenesis of GCM, and inhibitors of NETosis could alleviate inflammation in GCM, which could be applied as new targets for treatment.

Given the role of NETosis in GCM, we next wondered whether this process could be targeted therapeutically to ameliorate GCM in clinical practice. Observations of human heart specimens highlighted the potential clinical importance of this question. According to the 2013 ESC Task Force,^46^ we collected heart samples from patients with GCM and from patients with chronic heart failure with dilated cardiomyopathy (DCM) who had undergone HTx and compared them with those from healthy controls. The demographic data are presented (Supplementary material online, Table S4). Multifocal inflammatory infiltrates consisting of lymphocytes with multinucleated giant cells were observed in GCM (Figure 5D). We found that *MPO* and *H3CIT* were significantly expressed at higher levels in patients with GCM than in patients with DCM and healthy controls (Figure 5D). This result indicated that neutrophil infiltration was also detected and NETosis occurred in patients with GCM. Together, these data demonstrate the clinical relevance of NETosis to patients with GCM and may serve as a novel therapeutic target for clinical usage.

### Establishment of IMC analysis for the heart tissue of patients with GCM

Based on the IMC protocols for liver cancer and breast cancer,^47^^,^^48^ we established a 35-marker panel for heart tissue, including markers for endothelial cells, immune cells, and fibroblasts, the proliferation marker Ki-67, and the apoptotic marker cleaved caspase-3 (Supplementary material online, Figure S8 and Table S5).

**Figure 6:**
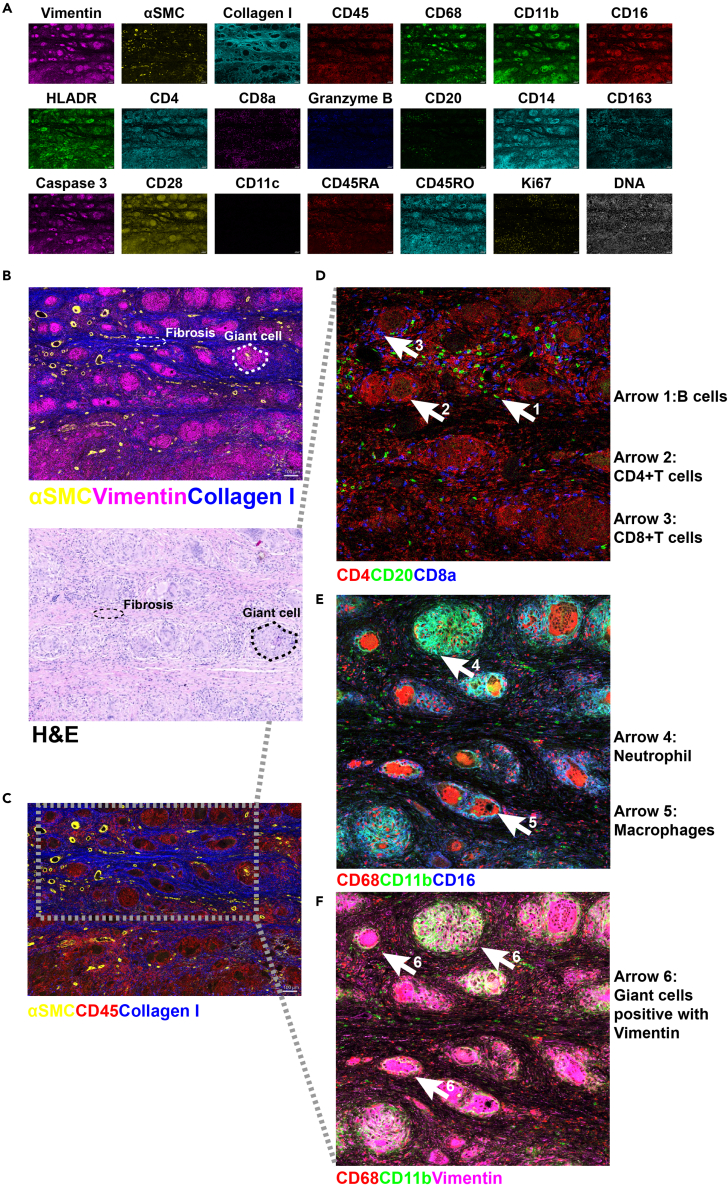
Representative mass cytometry images from a GCM heart sample were analyzed with the 35-marker panel Heart tissue from the patient with GCM was scanned by imaging mass cytometry (IMC) with a 35-marker panel. (A) Single-color staining of the indicated marker above each plot. (B) Vimentin (cyan), αSMA (yellow), and collagen I (blue) were used to portray the structure of heart tissue in the upper image. Multinucleate giant cells and fibrosis are highlighted with white dotted lines. In the lower image of H&E staining, the same structures are highlighted with black dotted lines. Scale bars, 100 μm. (C) αSMA (yellow), collagen I (blue), and CD45 (red) were used to highlight the inflammatory area. (D–F) Magnified views of the portal area selected by the dotted rectangle in C. D. CD4 (red), CD20 (green), and CD8 (blue) were used to specify lymphocyte clusters. E CD68 (red), CD11b (green), and CD16 (blue) were used to specify myeloid cell clusters. F. CD68 (red), CD11b (green), and vimentin (cyan) were used to indicate the features of multinucleated giant cells. Arrows 1: B cells (CD20^+^CD4^−^CD8a^−^), arrows 2: CD4^+^ T cells (CD4^+^CD8a^−^CD20^−^), arrows 3: CD8^+^ T cells (CD20^−^CD4^−^CD8^+^), arrows 3: neutrophils (CD11b^+^CD68^−^CD15^+^), arrows 4: neutrophils (CD11b^+^CD68^−^CD16^−^), arrows 5: macrophages (CD68^+^CD16^−^CD11b^−^), and arrows 6: giant cells positive for vimentin. See also Figure S8 and Table S5.

We analyzed heart tissues from patients with GCM and revealed 21 typical structural markers, such as collagen I and αSMC, and immunological markers, such as CD4 and CD20 (Figure 6A), among the 35 markers (Supplementary material online, Figure S8). Distinct histological features, including fibrosis (thin white dotted lines, revealed by collagen I staining of heart tissue) and multinucleate giant cells (thick white dotted line, characterized by vimentin staining), could be detected (Figure 6B). These features were consistent with those determined by H&E staining but were resolved in greater detail (Figure 6B). We detected major cell types, including *Cd45*^+^ immune cells and αSMA^+^ fibroblasts, and collagen I deposition in the extracellular matrix (Figure 6C). Notably, we found two patterns of multinucleate giant cell formation (Figure 6C): one kind was primarily composed of neutrophils, whereas the other was primarily composed of macrophages. Immune cell types within multinucleate giant cells included CD4^+^ T cells (2: CD4^+^CD8a^−^CD20^−^), CD8^+^ T cells (3: CD20^−^ CD4^−^ CD8^+^), neutrophils (4: CD11b^+^CD68^−^CD16^−^), and macrophages (5: CD68^+^CD16^−^CD11b^−^) (Figures 6D and 6E). B cells (1: CD20^+^CD4^−^CD8a^−^) were not a component of multinucleate giant cells (Figure 6D). Interestingly, all multinucleate giant cells were characterized by the expression of vimentin (Figure 6F). Vimentin is a citrullinated antigen in rheumatoid arthritis, which may underlie pathogenesis and be externalized during NETosis.^49^ Together, NETs were involved in the inflammatory responses in GCM.

## Discussion

Giant cell myocarditis is a rare autoimmune disease with a high mortality rate and recurrence rate after heart transplantation, and it was reported that this disease is a type of T cell lymphocyte-mediated inflammation of the myocardium that typically affects young and middle-aged adults.^50^ Although some clinical findings of this disease have helped to lead to a paradigm shift in the management of giant cell myocarditis resulting in an improvement in overall and transplant-free survival, such as combination immunosuppressive therapy, the immunological mechanism remains unknown. As scRNA-seq technology has been applied in the field of myocarditis,^19^ we thought it could help to uncover the immunological mechanism of GCM. This study could be a reference for us to gain insight into the immunological environment of GCM.

As we know, it is difficult to obtain fresh patient cardiac tissue samples, so we investigated them by the GCM model.^23^ According to this study, we found some results that were consistent with those of a previous study, and we also obtained some new findings about the pathogenesis of GCM. First, it was reported that most of the infiltrating immune cells in GCM were macrophages and T cells. In our study, we found that macrophages and T cells were the top 3 cell populations. Second, we found that neutrophils were the top 2 cell populations in GCM, which was the first single-cell report that neutrophils did appear in the GCM.^8^^,^^23^ Third, we further identified a subcluster of cells, such as *Arg1*^+^ macrophages and Th17 cells, that contributed to the pathogenesis of GCM. According to the immunological changes in GCM, innate immune cells received our attention, especially neutrophils. The recent RNA-seq study by Amancheria et al. was based on formalin-fixed paraffin-embedded human GCM heart samples, including 4 GCM, 3 lymphocytic myocarditis, 4 rejection, and 3 control samples.^51^ They found that pathway enrichment analysis in GCM showed that upregulated pathways were enriched for neutrophil degranulation, multiple cytokine signaling pathways, and phagocytosis, but they did not pay much attention to the neutrophil roles in GCM. In addition, the study did not focus on the immune cell types and immunological formation of giant cells. In our study, we analyzed the immune cell types and found that Th17 cells were the main T cell types in GCM, and neutrophils may contribute to the process of GCM. In addition, querying the Drug Gene Interaction Database identified several novel biologically plausible therapeutic targets, including *IL2RB*, *CD3G*, *CD3D*, *CD22*, and *CD20*. These targets were mostly associated with the specific immunity but not innate immunity. We first came up with the inhibition of NETosis to treat GCM. Together, our study on cardiac immune cells of GCM was more comprehensive than the RNA-seq study. In addition, we compared macrophage clusters observed in giant cell myocarditis in our report with the macrophage clusters from cardiac sarcoidosis in the report by Liu et al. due to the lack of single-cell data of giant cell myocarditis in the report by Liu et al.^52^ In the reports by Liu et al., spatial transcriptomics were reported in a myocarditis series that also included hearts from 3 patents with giant cell myocarditis, but not single-cell or single-nucleus data of giant cell myocarditis. We compared the results of the enrichment analysis (Supplementary material online, Figure S9). From the results, Macro_1 was proinflammatory. Macro_2 was similar to Mac_HLA, which was associated with antigen processing and presentation function. Macro_3 was similar to Mac_res, which is a resident macrophage that positively regulates the immune response. Macro_4 was similar to Mono, which was associated with the M2 process and recovery process. Macro_5 was similar to Mac_VCAN, which was related to the tumor necrosis factor signaling pathway, interferon signaling, and cytokine-mediated signaling pathway (Supplementary material online, Table S6). This result indicated that the macrophage cluster in GCM was similar to that in CS, but the detailed difference in single-cell levels between GCM and CS needs further investigation in the future.

GCM is a type of T cell-mediated autoimmunity, but it is not clear which kind of T cells contribute to the pathogenesis of GCM. Th17 cells have been reported in experimental autoimmune myocarditis and other autoimmune diseases.^19^^,^^53^^,^^54^ Th17 cells have been seen as candidate therapeutics for the treatment of autoimmune diseases.^55^ In the future, inhibitors that target Th17 cells could be investigated to determine whether they are helpful for patients with GCM.

Neutrophils are reported as the most abundant circulating leukocytes and the first line of defense against bacterial infections.^25^ Meanwhile, neutrophils also contribute to tissue damage during various autoimmune and inflammatory diseases. The intimate but complex involvement of neutrophils in various diseases makes them exciting targets for therapeutic intervention.^17^ It was also reported that NETosis may play a crucial role in inflammation and autoimmunity in a variety of autoimmune diseases, such as rheumatoid arthritis, systemic lupus erythematosus, and anti-neutrophil cytoplasmic antibody-associated vasculitis. NETosis can also activate other immune cells, such as B cells, antigen-presenting cells, and T cells. NETosis may be a central regulator of inflammation and autoimmunity, as well as a promising target for future therapeutics of inflammatory autoimmune diseases.^15^ In our study, we first demonstrated neutrophil infiltration into the myocardium of GCM rats and we performed a quantitative analysis of neutrophil infiltration that showed a significant increase in neutrophil infiltration in GCM rats compared to normal rats (Figure 4, Figure 5). Furthermore, we observed abundant NETosis in GCM myocardium (Figure 5C), which implied that NETosis might play roles in the pathogenesis of GCM. Then, we found that GSK484, an inhibitor of NETosis via PAD4, could ameliorate the inflammatory response of GCM, indicating that inhibiting NETosis could treat GCM rats (Figure 5B). In addition, both MPO and H3CIT expression also increased significantly in heart tissue samples of patients with GCM (Figure 5D), suggesting that neutrophils infiltrated into the hearts of patients with GCM and NETosis was also observed in patients with GCM. This result indicated that GSK484 may be used to treat patients with GCM in the clinic and should be further investigated in clinical trials in the future. Neutrophils make up nucleated giant cells that do not account for normal giant cell phagocytosis activity, and the mechanism of giant cell formation and phagocytosis activity requires further investigation. In general, bone marrow chimeric *Ldlr*-deficient mice reconstituted with either wild-type or *Pad4*-deficient cells were used to delineate the major role of *Pad4* as the main enzyme responsible for NETosis development and potentially the main therapeutic target.^56^ In the future, this approach should be applied to demonstrate that *Pad4*-dependent NETosis mechanisms are mainly involved in GCM pathophysiology in a rat model and that further strategies targeting PAD4 would be potentially relevant clinical options in patients with GCM. In addition, organoids represent a promising research model, helping us gain a more profound understanding of organs such as the intestine, brain, heart, and kidney.^57^ Due to the application of organoids in cardiovascular disease,^58^^,^^59^^,^^60^ organoid cell culture models would be of major interest to replace the proposed rat model in our group in the future. In addition, giant cells are thought to be involved in “cleaning up” and thus the appearance of neutrophils exhibiting phagocytic activity may not necessarily account for a pathogenic mechanism. The “cleaning up” role of neutrophils may be regarded as a pathogenic role of GCM. It was reported that neutrophils were quickly recruited to the ischemic region, where they initiate the inflammatory response, aiming at cleaning up dead cell debris.^61^ However, excess accumulation and/or delayed removal of neutrophils is deleterious. Neutrophils can promote myocardial injury by releasing reactive oxygen species, granular components, and proinflammatory mediators. Thus, the two-alternative hypothesis of neutrophils contributing into the pathogenesis of GCM includes NETosis and the “cleaning up” role of neutrophils.

Intercellular communication network analysis indicated that macrophages could induce NETosis by macrophage-derived *Il1β* (Supplementary material online, Figure S6A). In addition, we found that neutrophils were able to recruit immune cells, specifically T cells, into the heart muscle via the CXCL-CXCR3 signaling pathway. Macrophages were also involved in expressing CXCL chemokines (*Cxcl9*, *Cxcl10*, and *Cxcl11*) to attract CXCR3^+^ T cells.^62^ These CXCL chemokines were higher in macrophages in the GCM than in controls, suggesting that macrophages in the GCM induced T cells as well as neutrophils (Supplementary material online, Figure S7).

According to IMC analysis, all the multinucleated giant cells were positive for the expression of vimentin (Figure 6F). This suggests that vimentin may be an autoantigen extended by NETosis,^63^ which could induce many autoimmune diseases, such as lupus nephritis^64^ and rheumatoid arthritis.^49^ To back up this speculation, we will compare the difference in the level of vimentin autoantibodies in serum from GCM rats and GSK484-treated rats by ELISA or other methods in the future.

Among other cells, DC cells and NK cells were low in number (Supplementary material online, Table S2), but several genes with known functions were noted in DC cells, including *Tap1* and *Psmb9*, which have a role in antigen processing and presentation. For NK cells, we found that all NK cell cluster numbers were decreased in GCM compared with normal controls, suggesting that the roles of NK cells in GCM were very small. This result was different from those in viral myocarditis.

Recently, some single-cell RNA sequencing studies on viral myocarditis have been reported.^65^^,^^66^ The cell populations reported in viral myocarditis were roughly the same but also different. In viral myocarditis, we found that the major T cell subtypes were Th17 cells, CTLs, and Treg cells, which were similar to the T cell subtypes in GCM. However, the proportion of CTLs in GCM decreased, which was different from the increase in CTLs in viral myocarditis. For macrophages, we found that the transcriptomes of myeloid cells were mainly of the M2 phenotype in viral myocarditis, while the transcriptomes of macrophages were mainly of the M1 phenotype in GCM. Neutrophils participate in the pathogenic inflammatory and cardiac fibrosis process in viral myocarditis, with *Il1β* being the major driver of this process. Consistently, *Il1β* was also upregulated in neutrophils in GCM. This result indicated that myocarditis (viral myocarditis and giant cell myocarditis) is increasingly being considered an *Il1β*-mediated disease process so that neutralization of *Il1β* renders the mice more resistant to the development of myocarditis.^67^ However, NETosis was not reported in these viral myocarditis single-cell data,^65^^,^^66^ and the roles of NETosis need more investigation in viral myocarditis. Spatiotemporal transcriptomics has been applied in the study of viral myocarditis,^66^ but our study on GCM is lacking. In addition, single-cell RNA sequencing data of viral myocarditis include more cell types, including immune cells and nonimmune cells, which could help us to gain insight into the interaction between immune systems and other cell types to reveal the immune injury mechanism.

In summary, these data described the changes in the immunological environment of cardiac tissues from a GCM model using scRNA-seq. Some cell clusters were identified in the pathogenesis of GCM, such as macrophage and T cell subpopulations. NETosis was identified in GCM at the single-cell level. Furthermore, an inhibitor of NETosis alleviated immune cell infiltration in the GCM, which suggested that NETosis could be a potential candidate target for the treatment of GCM in the clinic.

### Limitations of the study

Although we have compared the differences in single-cell levels of macrophages between GCM and CS, we should emphasize two points. First, myocarditis and cardiac sarcoidosis are different diseases with different transcriptomic profiles, so it is not appropriate to compare macrophage clusters from cardiac sarcoidosis with those from our study. Last, these are also different species, as one is from the rat and the other from the human. Therefore, more investigations are needed to uncover the differences between the GCM rat model and the patient with GCM. In addition, the mechanism of antigen processing and presentation of antigen-presenting cells such as DCs in GCM needs more investigation, especially its mechanism of autoimmunity, which is also a limitation of this study. Many chemotactic pathways in neutrophils and macrophages were assumed to be generative and inconclusive, and additional investigations by inhibition of these chemotactic pathways are needed to confirm them, which is also a limitation.

## STAR★Methods

### Key resources table

**Table:** 

REAGENT or RESOURCE	SOURCE	IDENTIFIER
**Antibodies**		

Anti-ARG1 antibody	Abcam	Cat# ab92274,RRID:AB_10563668
Anti-C1QA antibody	Abcam	Cat# ab189922, RRID:AB_2894866
Anti-MPO antibody	Abcam	Cat# ab9535, RRID:AB_307322
Anti-H3cit antibody	Abcam	Cat# ab5103, RRID:AB_304752
Anti-CCDC25 antibody	Santa Cruz	Cat# sc-515201
Anti-CD45-BB515	BD Biosciences	Cat# 564590
7-AAD	BD Biosciences	Cat# 559925
For a list of antibodies used for analysis by Imaging mass cytometry (IMC), please see Table S5.	Various	Various

**Biological samples**		

Human heart tissue sample	Fuwai Hospital, Chinese Academy of Medical Sciences	N/A

**Chemicals, peptides, and recombinant proteins**		

Myosin heavy-chain-α (α-MyHC) peptide (Ac-RSLKLMATLFSTYASADR-OH)	DgPeptides	http://www.dgpeptides.com/Contactus.asp
Complete Freund’s adjuvant	Sigma	Cat# F5881
Mycobacterium tuberculosis H37 Ra	BD DIFCO	Cat# 231141
GSK484	MCE	Cat# HY-100514

**Critical commercial assays**		

Single-cell 5′ solution v2 reagent kit	Chromium	Cat# 1000006

**Deposited data**		

scRNA-seq processed data	This paper	Gene Expression Omnibus (GEO) database under accession code GEO: GSE221111.

**Experimental models: Organisms/strains**		

Rats: Lewis	Vital River Laboratories	https://www.vitalriver.com/#/animalModel/detailedReading?id=16&namecode=strain

**Software and algorithms**		

GraphPad Prism 8.3.1	GraphPad Software	RRID: SCR_002798
Image-Pro Plus software	MEDIA CYBERNETICS	RRID: SCR_007369
SPSS Statistics 26	SPSS, IBM	RRID: SCR_016479

### Resource availability

#### Lead contact

Further information and requests for resources and reagents should be directed to and will be fulfilled by the lead contact, Dr. Jiangping Song (fwsongjiangping@126.com).

#### Materials availability

This study did not generate new unique reagents.

### Experimental models and study participants detailsgcm model induction

Animal experiments were approved by the Animal Ethics Committee of Fuwai Hospital (No. FW-2022-0060) and this study also complied with the Declaration of Helsinki. Rats were humanely euthanized by administration of pentobarbitone sodium at 180 mg/kg at a concentration of 60 mg/mL via intraperitoneal injection. Seven-week-old female Lewis rats were purchased from Vital River Laboratories (Beijing, China), maintained in a specific pathogen-free facility, and provided free access to water and food. To induce the GCM model according to a previous report,^23^ the rats were subcutaneously injected with 1 mg of myosin heavy-chain-α (α-MyHC) peptide (Ac-RSLKLMATLFSTYASADR-OH; DgPeptides, Hangzhou, China) emulsified with complete Freund’s adjuvant (Sigma, F5881; 1:1, w/w) supplemented with Mycobacterium tuberculosis H37 Ra (BD DIFCO, No. 231141) on Days 0 and 7. The rats were examined using echocardiography and sacrificed on Day 21. Excised hearts were processed for immune cell isolation after histological examination.

#### Human heart sample collection

The use of human tissue in the present study was approved by the Human Ethics Committee of Fuwai Hospital, Chinese Academy of Medical Sciences. Written informed consent was obtained from each patient. Human heart samples were collected from patients who had undergone heart transplantation (HTx) in the operating room. The patients were divided into 2 groups: the GCM group (n = 3) and the chronic heart failure group (dilated cardiomyopathy [DCM] without myocarditis, n = 9). Healthy heart samples (n = 5) were obtained from brain-dead donors with a normal circulatory supply who were not suitable for transplantation due to technical or noncardiac reasons, such as body weight mismatch, according to the guidelines of China Transplant Services. All heart samples were obtained after fixation and fresh/frozen.

### Method details

#### Echocardiography and electrocardiogram (ECG) examination

Echocardiography was performed 3 days before the first immunization and 1 day before sacrifice. All rats were anesthetized with an intraperitoneal injection of ready-to-use anesthetic (isoflurane, 0.2 mL/10 g), and then the chest hair was removed. Each rat was placed and fixed in a dorsal position on a heated pad (37°C). Ultrasound gel was applied to the chest to place the electrodes. During the examination, the isoflurane concentration was reduced to the minimal amount (1–2%) required to achieve constant and comparable heart rates. M-mode images were acquired from the parasternal long axis and parasternal short axis to evaluate the morphology, including end-systolic and end-diastolic ventricular inner diameter of the left ventricle (LV), and cardiac functions, such as the ejection fraction and fractional shortening of the LV. Images were analyzed using the dedicated software package VevoLAB (Version 1.7.2).

#### Collection of heart tissues and histology

Surgically excised hearts were collected in Dulbecco’s Modified Eagle’s Medium (DMEM, Gibco, 11965-092) containing 10% fetal bovine serum (FBS) on ice. In each group, an approximately 1 mm-thick cross-section of the myocardium was removed from the middle of the heart and fixed with 4% paraformaldehyde overnight. Tissues were processed into paraffin sections and stained with hematoxylin and eosin. The remaining heart tissues were prepared as single-cell suspensions. Following published data,^68^ the heart tissue inflammatory condition was graded from 0 to 4: “0” means no inflammation; “1” means 1–5 distinct mononuclear inflammatory areas, with the involvement of 5% or less of the cross-sectional area of the heart; “2” means more than 5 distinct mononuclear inflammatory areas, or the involvement of over 5% but not over 20% of the cross-sectional area of the heart; “3” means profound mononuclear infiltration involving over 20% of the area, without necrosis; and “4” means diffuse inflammation with necrosis in the heart.

#### Preparation of the single cardiac cell suspension

Following the histological examination, we pooled 5 hearts from each group to isolate single cells. The remaining heart tissues were cut into small pieces in prechilled phosphate-buffered saline (PBS) and digested with 400 U/ml collagenase type II (Worthington, 43J14367B) in a 37°C water bath with mild shaking. The single-cell suspension was filtered with a 40 μm cell strainer and collected by centrifugation at 400 × g for 5 min. The supernatant was discarded, and the cell pellets were resuspended in 1 mL of 10% FBS/DMEM.

#### Flow cytometry and fluorescence-activated cell sorting (FACS)

The single-cell suspension was washed twice with PBS through centrifugation and resuspension. The cells were suspended in 500 μL of staining buffer containing anti-CD45-BB515 (BD Biosciences, Cat#564590) at a dilution of 1:200 per 10^6^ cells, incubated on ice for 20 min and then washed twice with PBS. Stained cells were then stained with a 1:20 dilution of 7-AAD (BD Biosciences, Cat#559925) and subjected to FACS. The prepared cells were analyzed using a FACS Aria II cell sorter (BD Biosciences). Then, viable leukocytes (7-AAD^-^ *Cd45*^+^) were sorted for further scRNA-Seq. Cell suspensions were incubated in low-absorbent microcentrifuge tubes on ice. Twenty microliters of the cell suspension (∼20,000 cells) was loaded on one Chromium Single-Cell Controller chip (10x Genomics).

#### scRNA-seq library preparation for 10x Genomics single-cell 5′ sequencing

Cardiac leukocyte suspensions were loaded on a Chromium Single-Cell Controller (10x Genomics) to generate a single-cell and gel bead emulsion.^69^ The scRΝΑ-seq libraries were prepared using a single-cell 5′ solution v2 reagent kit (Chromium, Cat#1000006) according to the protocol provided with the 10x Genomics Chromium Single-Cell Immune Profiling Solution.^70^ Briefly, FACS-sorted *Cd45*^+^ immune cells (90–95% viability) were encapsulated into droplets. Following the reverse transcription step, the droplets were disrupted, and the barcoded cDNAs were purified with DynaBeads and subjected to 14 cycles of polymerase chain reaction (PCR) amplification (98°C for 45 s; [98°C for 20 s, 67°C for 30 s, and 72°C for 1 min] x 14 cycles; 72°C for 1 min). The resulting amplified cDNAs were sufficient to construct 5′ gene expression libraries. The cDNAs from the single-cell transcriptomes (50 ng) were fragmented, subjected to two rounds of size selection with SPRI beads (average size 450 bp), and sequenced on an Illumina HiSeq Xten instrument (High Output V2 kit, 150 cycles).

#### Drug treatment

Other rat GCM models (n = 20) were established. Then, half of the GCM rats were administered GSK484 (4 mg/kg, MCE, Cat#HY-100514) in PBS via intraperitoneal injection. Rats received injections once daily for two weeks (from Day 8 to Day 20) before sacrifice (Figure 5A). Then, all the rats and the corresponding GCM treatment group (n = 5, per group) were sacrificed on Day 21, and the hearts were excised and processed for immune cell isolation and pathological examination.

#### Immunohistochemical (IHC) staining

IHC staining was performed using the reported protocol.^71^ Briefly, formaldehyde-fixed paraffin-embedded (FFPE) sections were dewaxed with methanol, subjected to antigen retrieval, blocked for 30 min, incubated with a 1:100 dilution of an anti-ARG1 antibody (Abcam, Cat# ab92274,RRID:AB_10563668) overnight at 4°C (anti-C1QA, Abcam, Cat# ab189922, RRID:AB_2894866, 1:100; anti-MPO, Abcam, Cat# ab9535, RRID:AB_307322, 1:100; anti-H3cit, Abcam, Cat# ab5103, RRID:AB_304752; anti-CCDC25, Santa Cruz, sc-515201,1:50), and then incubated with HRP-conjugated goat anti-mouse/rabbit IgG (ZSGB-BIO, Cat#PV-6002) at room temperature for 1 h. A DAB kit (ZSGB-BIO, Cat#ZLI-9019) was used for detection. The whole slide was scanned with an automatic digital slide scanning system (ZEISS, Axio Scan.Z1). The intensity of MPO and H3cit expression was measured with Image-Pro Plus software (RRID: SCR_007369).

#### Quantification of gene expression in single cells, determination of the major cell types, identification of marker genes, enrichment pathway analysis and cell‒cell interaction (CCI) analysis

Single-cell RNA-seq data were quantified using the 10x software package CellRanger 2.2.0 to map the data to the Rat (Rnor6) reference genome. We next removed the cells that (1) had greater than 10% expression originating from mitochondrial genes, (2) expressed less than 200 genes or greater than 5,000 genes, and (3) had less than 1,000 unique molecular identifier (UMI) counts or greater than 40,000 UMI counts to filter out low-quality cells and doublets (Supplementary material online, Table S1). Clustering was performed using a deep embedding algorithm for single-cell clustering (DESC) based on the variable genes identified with the filter_genes_dispersion function in Scanpy.^72^^,^^73^ DESC is an unsupervised deep learning-based clustering method for single-cell data. DESC iteratively learns cluster-specific gene expression signatures and cluster assignments to improve clustering accuracy and remove batch effects. First, DESC pretrains an autoencoder and initializes the clustering using Louvain. Then, the software iteratively fine-tunes the encoder and cluster layer to produce the final cluster assignment. We classified cells into different cell types using graph-based clustering on the informative principal components with the most appropriate clustering methods. This approach identified cell clusters that were readily assigned to known cell lineages according to the expression of marker genes. Finally, this strategy yielded 24 clusters, as listed in Supplementary material online, Table S2. The differentially expressed genes (DEGs) in each cluster were determined using the Wilcoxon rank-sum test implemented in Seurat 3.0 FindAllMarkers function.^74^ The genes with Bonferroni-adjusted p values less than 0.01 were considered DEGs for each cluster and subjected to subsequent analyses. A pathway enrichment analysis of each cluster was performed using Metascape (http://metascape.org/).^75^ CCI analysis was performed based on labeled GCM scRNA-seq data using CellChat.^41^

#### Reagents and antibodies

The compound GSK484 was purchased from MedChemExpress (MCE, Shanghai, China). The anti-CD45-BB515 antibody and 7-AAD viability staining solution were purchased from BD Biosciences (CA, USA). The anti-ARG1 antibody (Cat#ab92274, RRID:AB_10563668), anti-C1QA antibody (Cat#ab189922, RRID:AB_2894866), anti-MPO antibody (Cat#ab9535, RRID:AB_307322) and anti-H3cit antibody (Cat#ab5103, RRID:AB_304752) were purchased from Abcam (Cambridge, UK). Anti-CCDC25 antibody (Cat#sc-515201) was purchased from Santa Cruz (TX, USA).

#### Imaging mass cytometry (IMC) section preparation

FFPE heart tissue samples from patients with GCM were cut into 4 μm sections via the HistoCore MULTICUT system (Leica), and the samples were heated at 68°C for 1 h. Dewaxing was performed by incubating the sections in xylene at 68°C for 10 min twice. Then, the sections were rehydrated for 5 min each in 95%, 85% and 75% ethanol at room temperature (RT), followed by heat-mediated antigen retrieval for 30 min at 100°C in sodium citrate solution. After natural cooling to RT, the sections were washed twice with PBS containing 0.5% Tween 20 and 1% bovine serum albumin (PBS-tris buffer (TB)) for 5 min each time. Then, section blocking was carried out with SuperBlock (Thermo Fisher Scientific, Cat#37515) for 30 min at RT. After three washes in PBS-TB, the sections were incubated with an antibody cocktail at 4°C overnight. The antibodies were purchased from Fluidigm (Supplementary material online, Table S5). The final product panel is presented in the Supplementary material online, Figure S6. After antibody cocktail incubation overnight at 4°C, three additional PBS-TB washes were performed. To label cell nuclei, the sections were incubated with an intercalator-Ir (Fluidigm, Cat#201192B) solution in PBS-TB (1.25 μM) for 30 min at RT, followed by two washes with PBS-TB and one wash with ddH_2_O.^48^

#### IMC analysis

An imaging mass cytometer (Fluidigm, Hyperion) was used to scan the tissue sections to generate multiplexed images. To segment image data into single-cell data, we used CellProfiler software (the Whitehead Institute for Biomedical Research and MIT’s CSAIL) to obtain mask files.

### Quantification and statistical analysis

Multiple group comparisons were made by one-way ANOVA followed by the Bonferroni test.n refers to the sample size. A value of p < 0.05 was considered statistically significant. ∗p < 0.05; ∗∗p < 0.01; ∗∗∗p < 0.001; ∗∗∗∗p < 0.0001; ns, not significant. Data are expressed as mean ± SEM. Statistical analysis was performed with GraphPad Prism 8.3.1 (Graphpad Software, USA, RRID: SCR_002798) and SPSS Statistics 26 (IBM, USA, RRID: SCR_016479).

## Data Availability

• The scRNA-seq data have been deposited into the Gene Expression Omnibus (GEO) database under accession code GEO: GSE221111. • All the codes used in the manuscript are deposited in GitHub (git@github.com:moxiuxue/GCM.git[github.com]). • Data, analytical methods, and study materials will be made available to other researchers upon request from the lead contact for the purpose of reproducing results or replicating procedures.
