# Supplementary material for: Inhibition of NETosis via PAD4 alleviated inflammation in giant cell myocarditis
Source: iScience. 2023 Jun 16;26(7):107162. doi: 10.1016/j.isci.2023.107162 (PMC10391931; doi:10.1016/j.isci.2023.107162)
Supplement: Document S1. Figures S1–S9 and Tables S1–S6 [file mmc1.pdf]

iScience, Volume *26*

Supplemental information

Inhibition of NETosis via PAD4 alleviated inflammation in giant cell myocarditis

Zhan Hu, Xiumeng Hua, Xiuxue Mo, Yuan Chang, Xiao Chen, Zhenyu Xu, Mengtao Tao, Gang Hu, and Jiangping Song

Supplemental Information

Supplementary Tables:

Table S1. Related to Figure 1B. The cell number in the study before/after QC.

Table S2. Related to Figure 1B. The transcriptional condition and percentage of cell clusters of immune cells.

Table S3. Related to Figure 2D. The associated genes which were used in macrophage analysis.

Table S4. Related to Figure 5C. Baseline characteristics of GCM and DCM.

Table S5. Related to Figure 6A. Imaging mass cytometry antibody panel.

Table S6. Related to Figure 2A and 2C. Compare macrophages between cardiac sarcoidosis and giant cell myocarditis.

Supplementary Figures:

Figure S1. Related to Figure 1A. The phenotype of GCM.

Figure S2. Related to Figure 1A. The live immune cell percentage derived by flow cytometry analysis.

Figure S3 Related to Figure 1B. The gene and UMI distributions in CTL and GCM.

Figure S4. Related to Figure 1B. Feature plots of hallmark genes to identify the cell types.

Figure S5. Related to Figure 2A. Feature plots of hallmark genes to identify the macrophages origin.

Figure S6. Related to Figure 4D. Intercellular communication network analysis revealed that the roles of NETosis in GCM.

Figure S7 Related to Figure 4D. CXCL chemokines expression in cell clusters from different groups.

Figure S8 Related to Figure 6A. Single color staining of 35-marker panel.

Figure S9 Related to Figure 2A and 2C. Compare macrophages clusters between GCM and CS.

1 **Table S1. The cell number in the study before/after QC**

Groups	Expression library detected cells	Discard cell number QC	Cells number after QC	Proportion of total cells in analysis
CTL	9,046	480	8,566	54.51%
GCM	7,786	638	7,148	45.49%
Sum	16,832	1,118	15,714	100.00%

2

3 **Table S2. The transcriptional condition and percentage of cell clusters of immune cells**

Cluster ID	Cell Type	CTL	GCM	Average nGene	Average nUMI
0	Neutrophils (Neu_1)	1.39%	98.61%	766	2,598
1	NK Cells (NK_1)	94.99%	5.01%	1,733	5,913
2	Macrophage (Macro_1)	0.36%	99.64%	2,968	16,935
3	Macrophage (Macro_2)	4.56%	95.44%	3,435	19,961
4	Macrophage (Macro_3)	94.99%	5.01%	1,635	4,690
5	Neutrophils (Neu_2)	71.39%	28.61%	850	2,826
6	T Cells (T_1)	97.77%	2.23%	1,383	4,316
7	Macrophage (Macro_4)	84.84%	15.16%	2,261	9,710
8	Macrophage (Macro_5)	24.61%	75.39%	2,213	10,842
9	T Cells (T_2)	20.23%	79.77%	1,824	6,651
10	Neutrophils (Neu_3)	0.00%	100.00%	735	3,122
11	T Cells (T_3)	81.66%	18.34%	1,524	5,331
12	cDC	88.70%	11.30%	2,805	14,764
13	B Cells	98.46%	1.54%	1,163	3,036
14	DC	13.33%	86.67%	2,768	15,183

15	NK Cells (NK_2)	95.94%	4.06%	1,731	5,505
16	NK Cells (NK_3)	100.00%	0.00%	1,933	7,376
17	cDC	61.19%	38.81%	3,229	18,914
18	Lymphoid progenitors	74.23%	25.77%	3,152	15,102
19	Endothelial	95.42%	4.58%	2,121	6,898
20	T Cells (T_4)	19.27%	80.73%	2,232	9,069
21	Granulocyte-macrophage progenitors	77.91%	22.09%	3,516	18,551
22	ILC2	96.47%	3.53%	1,433	5,165
23	pDC	25.68%	74.32%	3,000	11,409
24	Fibroblasts	89.13%	10.87%	2,065	6,956

Table S3. The associated genes which were used in macrophage analysis

ISG scores			Phagocytosis	Inflammation
STAT1	IDO1	BATF2	P2Y2	CHUK
FBXO6	GBP6	IFI44L	CX3CR1	FADD
PARP10	STAT2	XAF1	GPR132	IKBKB
OAS2	TIMM10	OASL	LRP1	IKBKG
TRIM22	STAT1	IFI44	CD14	IL1A
PARP10	PARP12	OAS2	CD36	IL1R1
GBP3	PARP9	TRIM6	BAI1	IRAK1
ZNF684	GBP1	HES4	TIMD4	MAP3K1
CARD17	GBP4	OTOF	STAB1	MAP3K14
GALM	IFIH1	IFI44L	MERTK	MAP3K7
DHX58	IRF7	DDX60	AXL	MYD88
CEACAM1	PARP14	IFITM3	CD209	NFKB1
UBE2L6	IFIT2	IFIT3	CD163	NFKBIA

CEACAM1	IFI35	CXCL10	NR1H3	RELA
APOL6	STAT1	SERPING1	MSR1	RIPK1
SOCS1	IFIT5	SPATS2L	CD169	TAB1
DDX58	ZNFX1	RSAD2	GULP1	TLR4
UBE2L6	PHF11	LY6E	ELMO1	TNF
TNFAIP6	ACTA2	OAS1	ITGAV	TNFAIP3
REC8	SP140	IFIT1	ITGB5	TNFRSF1A
RNF213	ABCA1	IFIT3	DOCK1	TNFRSF1B
ISG20	TCN2	OAS3	RAC	TRADD
DYNLT1	ZC3HAV1	OAS1	RHOG	TRAF6
TRIM56	HSH2D	OASL	LAMP1	
SP140	GBP2	CMPK2	LAMP2	
TRIM38	TRIM5	HERC5	RAB7	
PSMB9	RHBDF2	OAS1	RAB5	
CPT1B	TMEM140	MX1	SIGLEC1	
BST2	ADAR	4-Sep	ITGAM	
NMI	BTN3A1	HERC6	ITGAX	
RBCK1	LGALS9	IFITM1	ITGA5	
TRAFD1	NBN	TRIM5	CLEC7A	
TRIM21	TYMP	EIF2AK2	SCARF1	
CARD16	SRBD1	AIM2	C1QA	
CHMP5	NCOA7	MT1A	C1QB	
TAP2	UNC93B1	MOV10	C1QC	
SP110	SP100	CCL8	CALR	
GADD45B	DHRS9	HELZ2	C1QR	
TAP1	TRANK1	ZBP1	FCGR1A	
TRIM5	WARS		FCGR2A	

NT5C3A	LAP3		FCGR2B	
ASPRV1	TNFSF10		FCGR3A	
IRF9	GBP1		FCGR3B	

Table S4. Baseline characteristics of GCM and DCM

**Characteristics**	**GCM**	**DCM**	**P value**
Number	3	9	
Demography			
Male（No. %）	3（100%）	5（55.6%）	0.49
Age（year）	42.3±7.2	25.9±17.1	0.28
BMI	24.9±2.5	20.0±2.5	0.01
Clinical characteristics			
Diagnosis to transplantation(months)	6.7±3.1	66.1±90.6	0.28
Cardiac function			
Ⅰ	0（0%）	0（0%）	-
Ⅱ	1（33.3%）	2（22.2%）	1.00
Ⅲ	1（33.3%）	3（33.3%）	1.00
Ⅳ	1（33.3%）	4（44.4%）	1.00
SBP（mmHg）	102.7±24.0	92.78±12.8	0.60
Heart rate	92.3±10.5	89±11.0	0.73
History of viral infections			
virus infection within 6 months	1（33.3%）	3（33.3%）	1
Eosinophil percentage change			
Up	0（0%）	0（0%）	-
Down	2（66.7%）	0（0%）	0.045
Autoimmune Diseases	1（33.3%）	0(0%)	1
ECG			

AF	1（33.3%）	4（44.4%）	1.00
LBBB	0（0%）	0（0%）	-
PVT	2（66.7%）	4（44.4%）	1.00
UCG			
LAD（mm）	43.0±7.9	41.3±5.1	1.00
LVEDD（mm）	66.0±7.6	68±4.4	0.63
EF（%）	27.7±7.5	26.3±7.2	0.01
MR	2（66.7%）	7（77.7%）	1.00

GCM: giant cell myocarditis；DCM: dilated cardiomyopathy; BMI: body mass index; ECG: electrocardiogram; AF: atrial fibrillation; LBBB: left bundle branch block; PVT: paroxysmal ventricular tachycardia; UCG: ultrasonic cardiogram; LAD: left atrial diameter; LVEDD: Left ventricular end diastolic diameter; EF: Ejection fraction; MR: mitral regurgitation (moderately to severe).

Table S5. Imaging mass cytometry antibody panel

Cat#	Antibody (clone)	Metal label information	Source
3141017D	Smooth Muscle Actin (1A4)	141Pr	Fluidigm
3142014D	CD19 (6OMP31)	142Nd	Fluidigm
3143027D	Vimentin (D21H3)	143Nd	Fluidigm
3144025D	CD14 (EPR3653)	144Nd	Fluidigm
3145015D	T-bet/TBX21 (D6N8B)	145Nd	Fluidigm
3146020D	CD16 (EPR16784)	146Nd	Fluidigm
3147021D	CD163 (EDHu-1)	147Sm	Fluidigm
3148021D	CD278/ICOS (D1K2T)	148Nd	Fluidigm
3149028D	CD11b (EPR1344)	149Sm	Fluidigm
3150031D	PD-L1 (E1L3N)	150Nd	Fluidigm
3151026D	IgM (MHM-88)	151Eu	Fluidigm
3152016D	CD45 (CD45-2B11)	152Sm	Fluidigm

3153028D	CD223/Lug-3 (Dug40)	153Eu	Fluidigm
3154026D	CD11c (3.9)	154Sm	Fluidigm
3155016D	FoxP3 (236A/E7)	155Gd	Fluidigm
3156033D	CD4 (EPR6855)	156Gd	Fluidigm
3158031D	CD73 (EPR6115)	158Gd	Fluidigm
3159035D	CD68 (KP1)	159Tb	Fluidigm
3160026D	CD28 (YTH913.12)	160Gd	Fluidigm
3161029D	CD20 (H1)	161Dy	Fluidigm
3162035D	CD8a (D8A8Y)	162Dy	Fluidigm
3163030D	CD196/CCR6 (G034E3)	163Dy	Fluidigm
3164027D	Arginase-1 (D4E3M)	164Dy	Fluidigm
3165041D	CD279/PD-1 (EH12.2H7)	165Ho	Fluidigm
3166031D	CD45RA (HI100)	166Er	Fluidigm
3167021D	granzyme B (EPR20129-217)	167Er	Fluidigm
3168022D	Ki-67 (B56)	168Er	Fluidigm
3169023D	Collagen Type I (Polyclonal)	169Tm	Fluidigm
3170022D	CD3 (UCHT1)	170Er	Fluidigm
3171025D	CD27 (LG.3A10)	171Yb	Fluidigm
3172027D	Cleaved Caspase 3 (5A1E)	172Yb	Fluidigm
3173016D	CD45RO (UCHL1)	173Yb	Fluidigm
3174025D	HLA-DR (LN3)	174Yb	Fluidigm
3175036D	CD25 (EPR6452)	175Lu	Fluidigm
3176026D	Perforin (dG9)	176Yb	Fluidigm

Table S6. Compare macrophages between cardiac sarcoidosis and giant cell myocarditis.

Cell cluster	Cell number	Maker genes	Major function	Peak phase	Corresponding to cardiac

(Naming in GCM)					sarcoidosis (CS)
Macro_1	1646	Prdx1, Prdx5, Ass1, Arg1	inflammatory response, antigen processing and presentation	GCM	
Macro_2	922	Ms4a7, Tmem176a, Tmem176b	antigen processing and presentation of exogenous peptide antigen via MHC class II	GCM	Mac_HLA
Macro_3	1077	Nr4a1	regulation of cell activation, positive regulation of cell death	CTL	Mac_res
Macro_4	798	Pf4	inflammatory response, response to type II interferon	CTL	Mono
Macro_5	841	Fcnb, Plac8, Vcan	Inflammatory response, response to IFN-β	GCM	Mac_VCAN

Supplemental Figures and legends

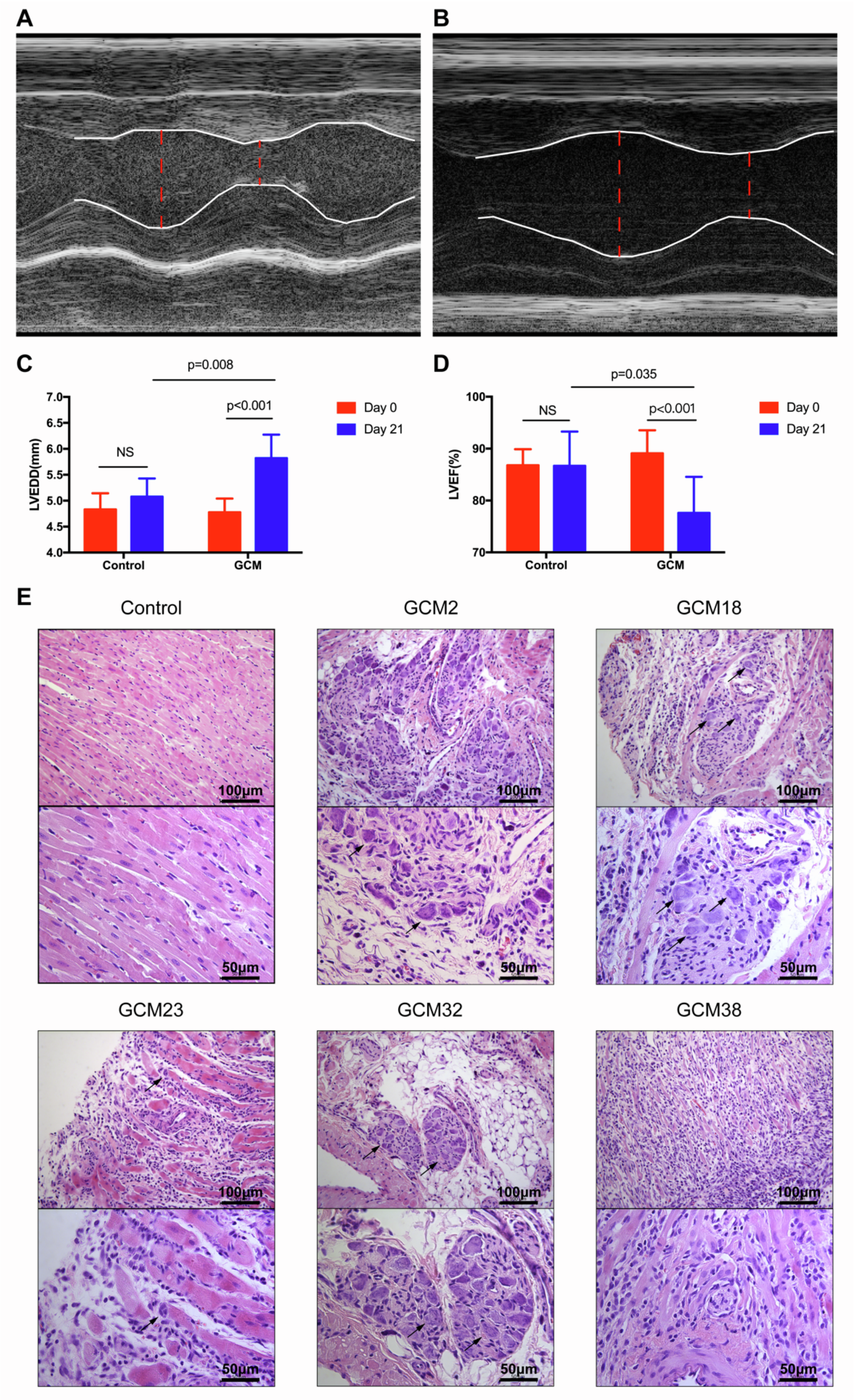

Figure S1. The phenotype of GCM. A-B. The short axis performance of control (A) and

GCM (B). C-D. Statistical analysis of LVEDD (C) and LVEF(D) between GCM and normal group. E. The typical histological performance of the GCM model. LVEF: left ventricular ejection fraction; LVEDD: left ventricular end-diastolic dimension. n = 5, ns, not significant, One-way ANOVA. Data are presented as mean ± SEM.

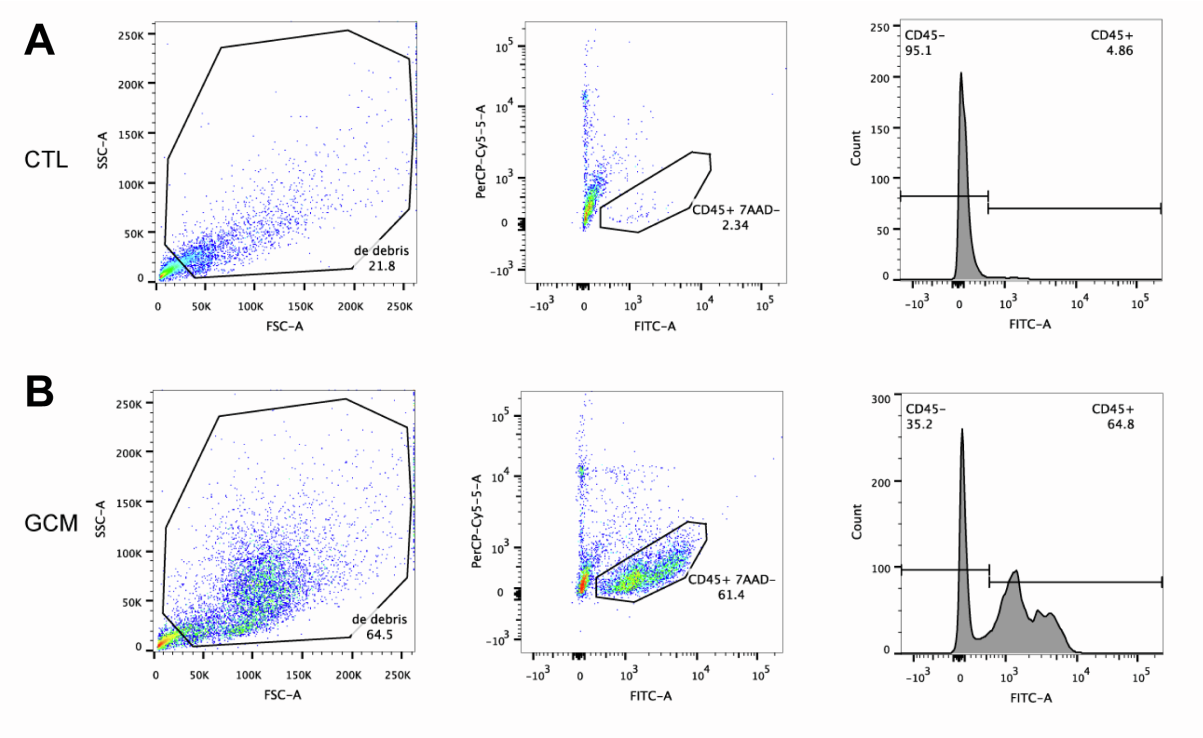

Figure S2. The live immune cell percentage derived by flow cytometry analysis. A-B. The gating strategy to sort live immune cells and the percentage of immune cells of CTL (A) and GCM (B). CTL: control; GCM: giant cell myocarditis.

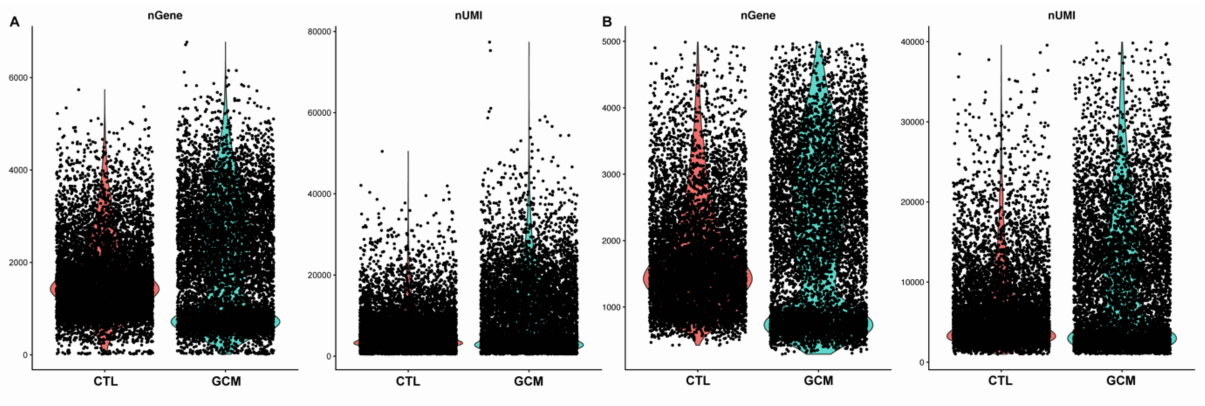

Figure S3. The gene and UMI distributions in CTL and GCM. A. The gene number distribution (left) and UMI count number distribution (right) of immune cells in CTL and GCM before quality control. B. The gene number distribution (left) and UMI count number distribution (right) of immune cells in CTL and GCM after quality control. UMI: unique molecular identifier.

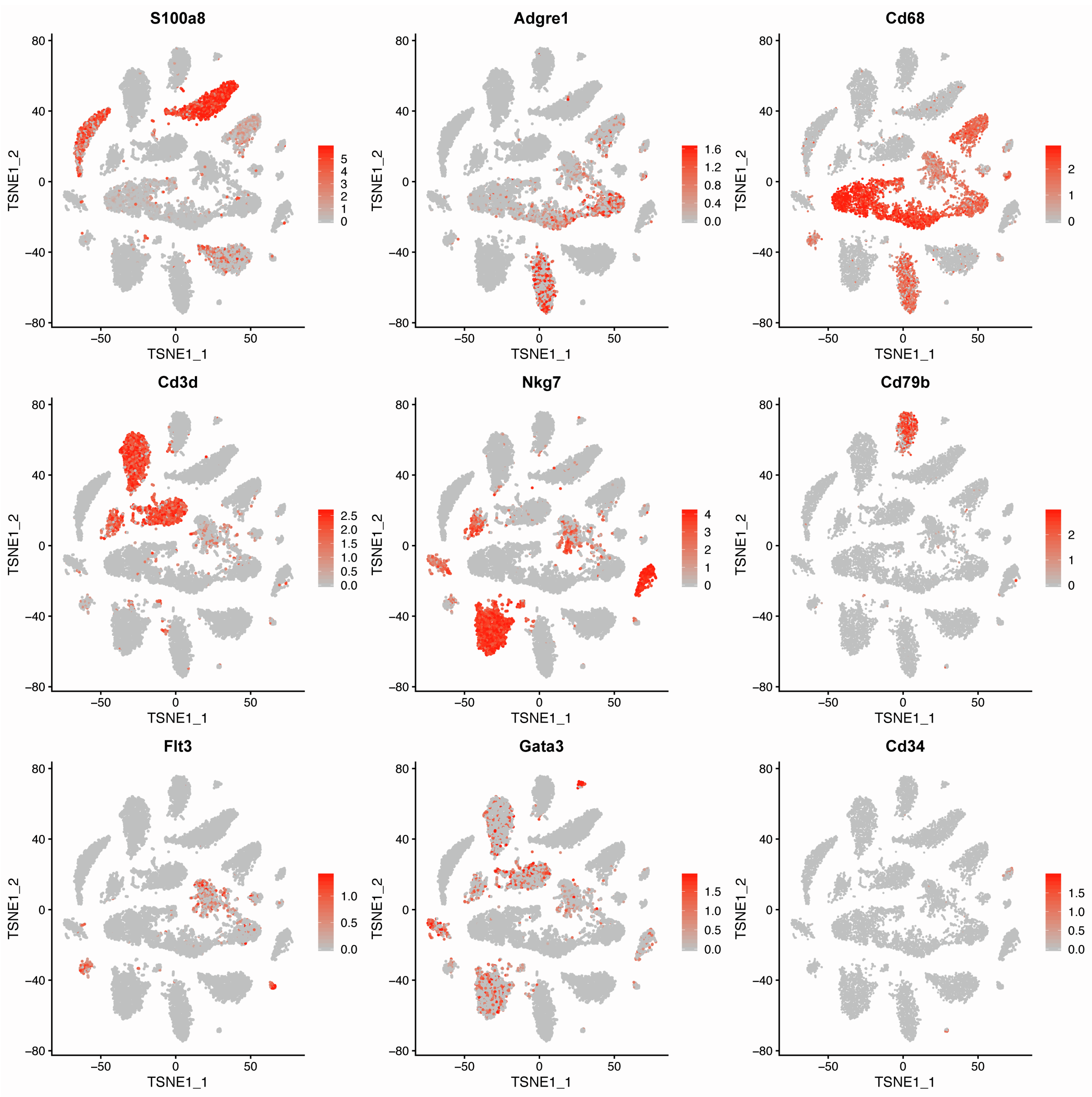

Figure S4. Feature plots of hallmark genes to identify the cell types.

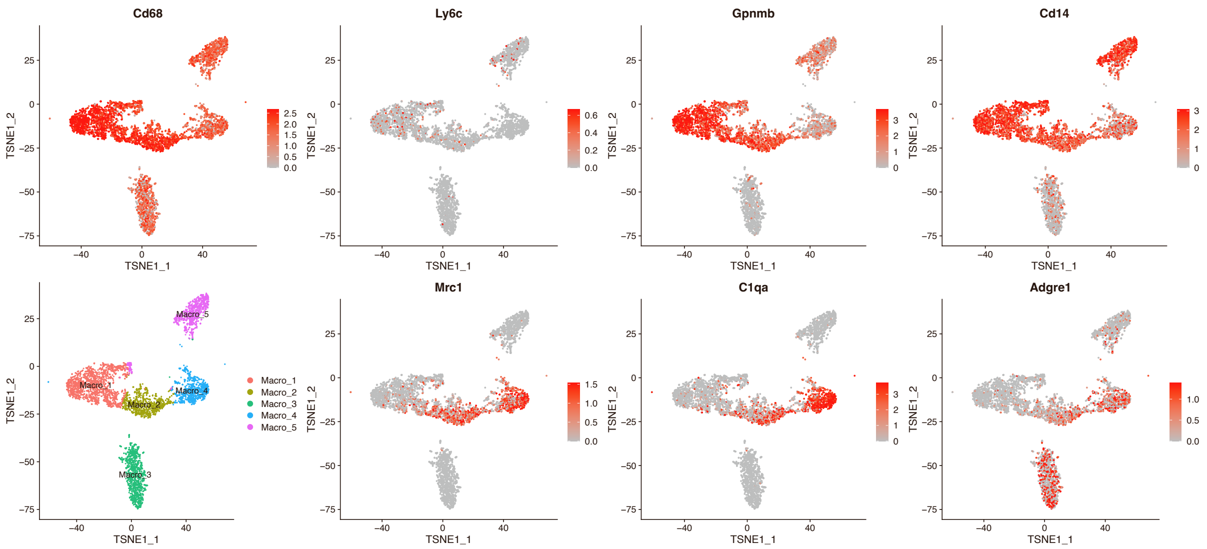

Figure S5. Feature plots of hallmark genes to identify the macrophages origin.

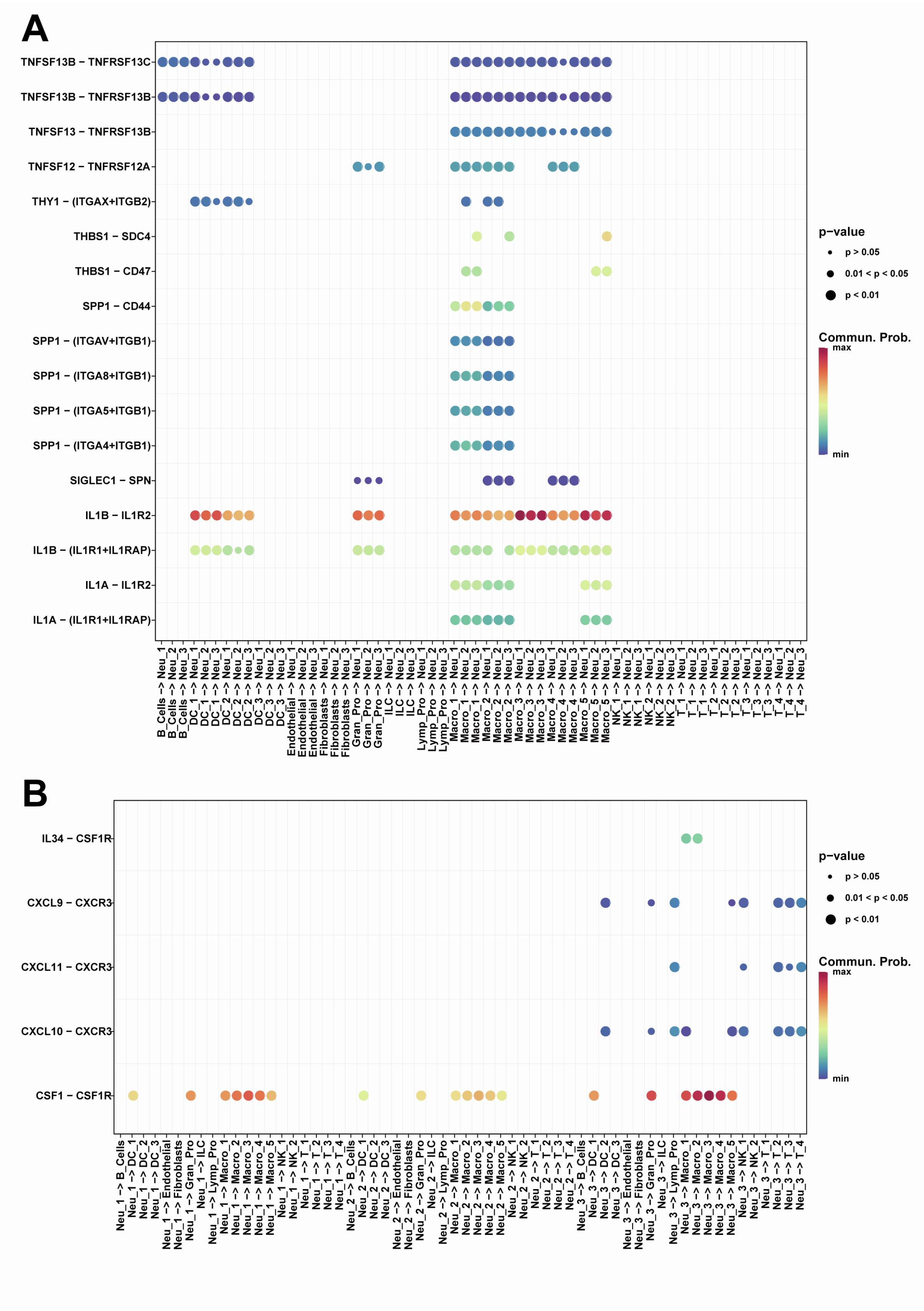
A
TNFSF13B – TNFRSF13C
TNFSF13B – TNFRSF13B
TNFSF13 – TNFRSF13B
TNFSF12 – TNFRSF12A
THY1 – (ITGAX+ITGB2)
THBS1 – SDC4
THBS1 – CD47
SPP1 – CD44
SPP1 – (ITGAV+ITGB1)
SPP1 – (ITGA8+ITGB1)
SPP1 – (ITGA5+ITGB1)
SPP1 – (ITGA4+ITGB1)
SIGLEC1 – SPN
IL1B – IL1R2
IL1B – (IL1R1+IL1RAP)
IL1A – IL1R2
IL1A – (IL1R1+IL1RAP)
p−value
p > 0.05
0.01 < p < 0.05
p < 0.01
Commun. Prob.
max
min
B
IL34 – CSF1R
CXCL9 – CXCR3
CXCL11 – CXCR3
CXCL10 – CXCR3
CSF1 – CSF1R
p−value
p > 0.05
0.01 < p < 0.05
p < 0.01
Commun. Prob.
max
min

Figure S6. Intercellular communication network analysis revealed that the roles of NETosis in GCM. A. From the source sets to the target sets (Neu_1, Neu_2, Neu_3). B. From the source sets (Neu_1, Neu_2, Neu_3) to the target sets.

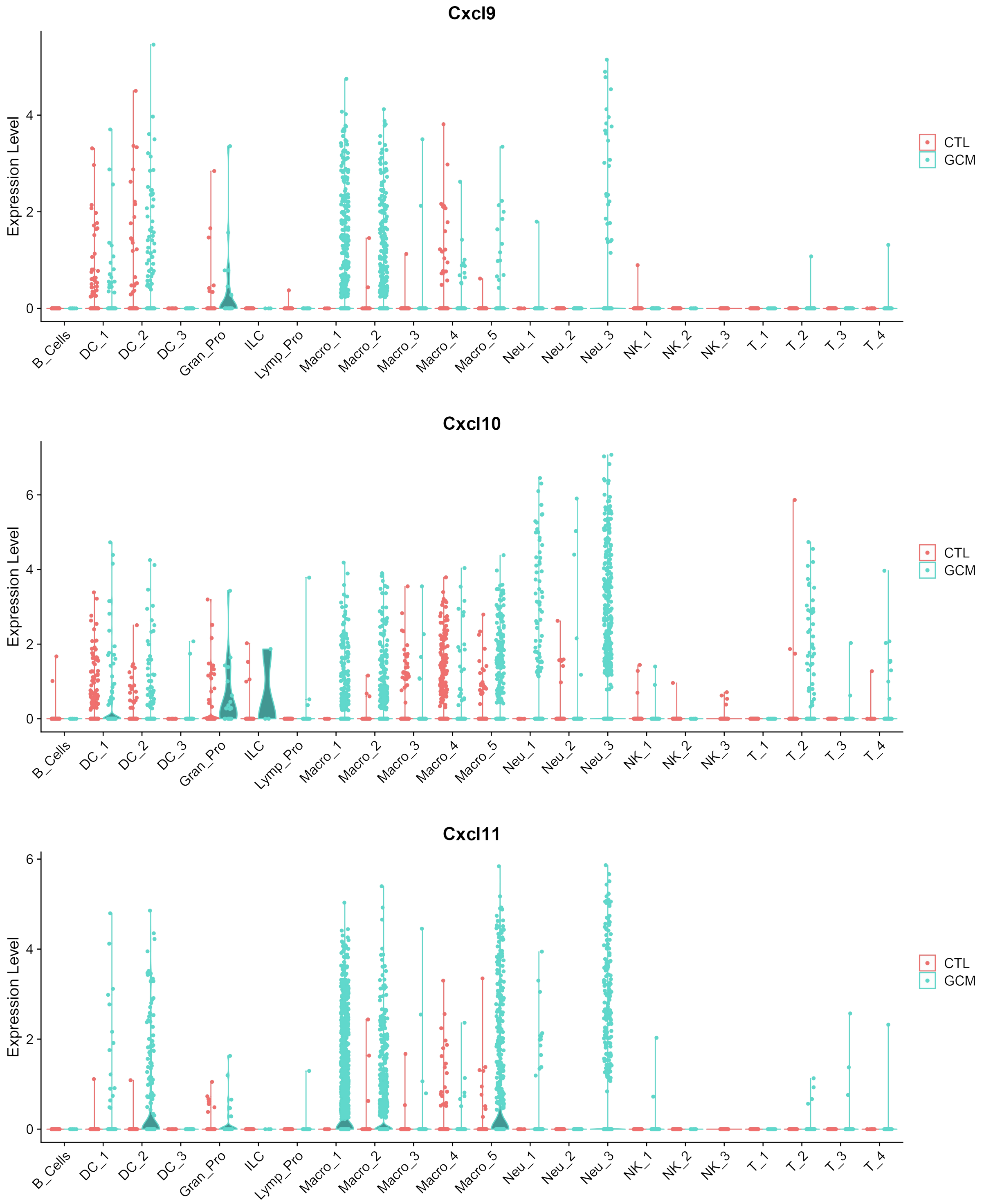

Figure S7. CXCL chemokines expression in cell clusters from different groups.

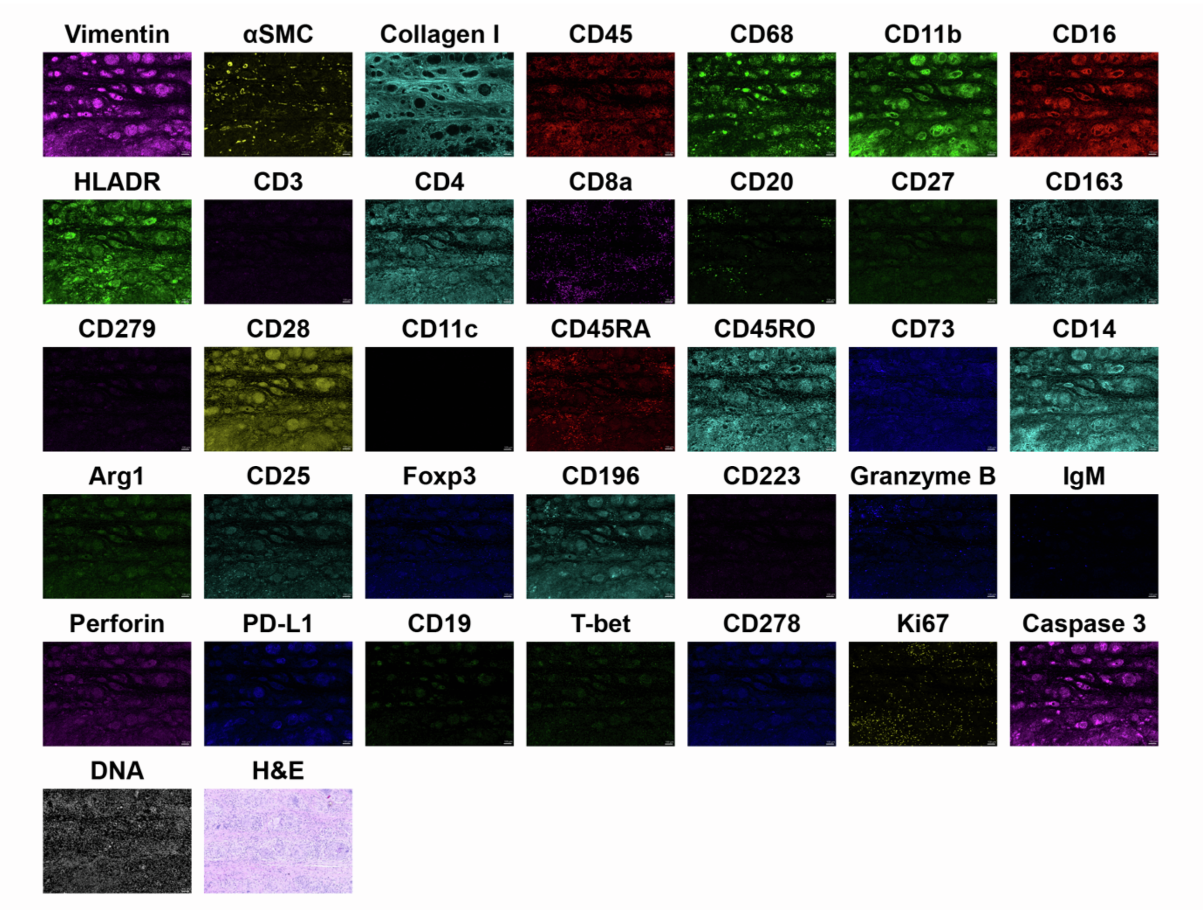

Figure S8. Single color staining of 35-marker panel. Heart tissue from the GCM patient was scanned by IMC with a 35-marker panel. The same region as Figure 6 with indicated marker above each plot.

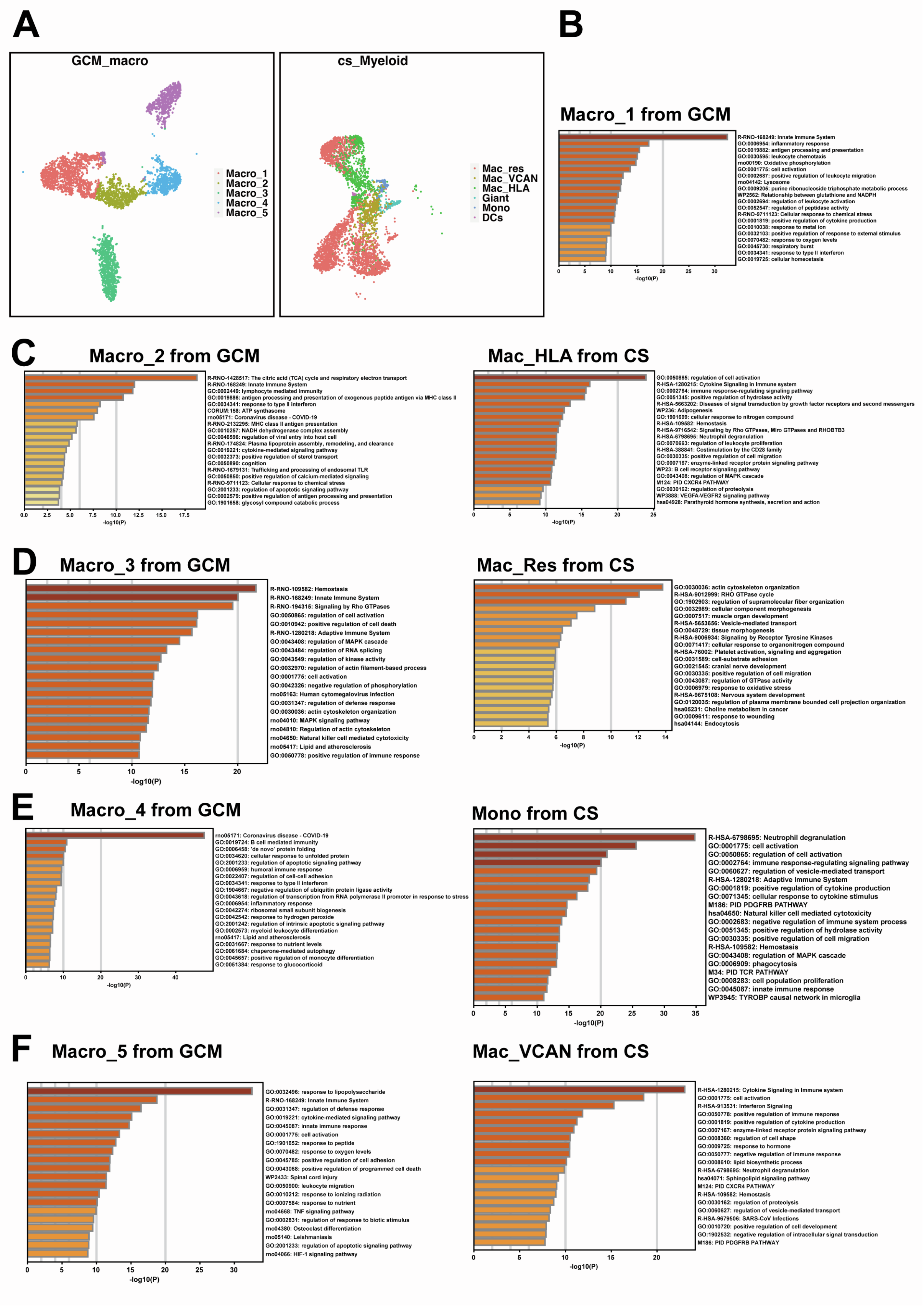

Figure S9. Compare macrophages clusters between GCM and CS. A. The UMAP projection of five macrophage clusters of our giant cell myocarditis (GCM) andsix myeloid

cell clusters of cardiac sarcoidosis (CS). B. The enrichment result of Macro_1. C. The enrichment results of Macro_2 and Mac_HLA. D. The enrichment results of Macro_3 and Mac_res. E. The enrichment results of Macro_4 and Mono. F. The enrichment results of Macro_5 and Mac_VCAN.
